# Blood culture utilization and epidemiology of antimicrobial-resistant bloodstream infections before and during the COVID-19 pandemic in the Indonesian national referral hospital

**DOI:** 10.1186/s13756-022-01114-x

**Published:** 2022-05-19

**Authors:** Robert Sinto, Khie Chen Lie, Siti Setiati, Suhendro Suwarto, Erni J. Nelwan, Dean Handimulya Djumaryo, Mulya Rahma Karyanti, Ari Prayitno, Sumariyono Sumariyono, Catrin E. Moore, Raph L. Hamers, Nicholas P. J. Day, Direk Limmathurotsakul

**Affiliations:** 1 Division of Tropical and Infectious Diseases, Department of Internal Medicine, Cipto Mangunkusumo National Hospital - Faculty of Medicine Universitas Indonesia, Jakarta Pusat, DKI Jakarta, 10430 Indonesia; 2 Faculty of Medicine Universitas Indonesia, Jakarta Pusat, DKI Jakarta, 10440 Indonesia; 3 Infection and Antimicrobial Resistance Control Committee, Cipto Mangunkusumo National Hospital, Jakarta Pusat, DKI Jakarta, 10430 Indonesia; 4 Nuffield Department of Medicine, Centre for Tropical Medicine and Global Health, University of Oxford, Oxford, OX3 7LG UK; 5 Department of Internal Medicine, Cipto Mangunkusumo National Hospital, Jakarta Pusat, DKI Jakarta, 10430 Indonesia; 6 Faculty of Medicine Universitas Indonesia, Center for Clinical Epidemiology and Evidence Based Medicine, Cipto Mangunkusumo National Hospital, Jakarta Pusat, DKI Jakarta, 10430 Indonesia; 7 Department of Clinical Pathology, Cipto Mangunkusumo National Hospital, Jakarta Pusat, DKI Jakarta, 10430 Indonesia; 8 Department of Child Health, Cipto Mangunkusumo National Hospital, Jakarta Pusat, DKI Jakarta, 10430 Indonesia; 9 Director of Medical Service and Nursing, Board of Directors, Cipto Mangunkusumo National Hospital, Jakarta Pusat, DKI Jakarta, 10430 Indonesia; 10 Centre for Neonatal and Paediatric Infection, St George’s, University of London, Cranmer Terrace, London, SW17 0RE UK; 11 Eijkman-Oxford Clinical Research Unit, Jakarta Pusat, DKI Jakarta, 10430 Indonesia; 12 Mahidol Oxford Tropical Medicine Research Unit, Faculty of Tropical Medicine, Mahidol University, Bangkok, 10400 Thailand; 13 Department of Tropical Hygiene, Faculty of Tropical Medicine, Mahidol University, Bangkok, 10400 Thailand

**Keywords:** Antimicrobial resistance, Blood culture, Blood culture utilization, Bloodstream infection, COVID-19, Indonesia

## Abstract

**Background:**

There is a paucity of data regarding blood culture utilization and antimicrobial-resistant (AMR) infections in low and middle-income countries (LMICs). In addition, there has been a concern for increasing AMR infections among COVID-19 cases in LMICs. Here, we investigated epidemiology of AMR bloodstream infections (BSI) before and during the COVID-19 pandemic in the Indonesian national referral hospital.

**Methods:**

We evaluated blood culture utilization rate, and proportion and incidence rate of AMR-BSI caused by WHO-defined priority bacteria using routine hospital databases from 2019 to 2020. A patient was classified as a COVID-19 case if their SARS-CoV-2 RT-PCR result was positive. The proportion of resistance was defined as the ratio of the number of patients having a positive blood culture for a WHO global priority resistant pathogen per the total number of patients having a positive blood culture for the given pathogen. Poisson regression models were used to assess changes in rate over time.

**Results:**

Of 60,228 in-hospital patients, 8,175 had at least one blood culture taken (total 17,819 blood cultures), giving a blood culture utilization rate of 30.6 per 1,000 patient-days. A total of 1,311 patients were COVID-19 cases. Blood culture utilization rate had been increasing before and during the COVID-19 pandemic (both *p* < 0.001), and was higher among COVID-19 cases than non-COVID-19 cases (43.5 vs. 30.2 per 1,000 patient-days, *p* < 0.001). The most common pathogens identified were *K. pneumoniae* (23.3%), *Acinetobacter* spp. (13.9%) and *E. coli* (13.1%). The proportion of resistance for each bacterial pathogen was similar between COVID-19 and non-COVID-19 cases (all *p* > 0.10). Incidence rate of hospital-origin AMR-BSI increased from 130.1 cases per 100,000 patient-days in 2019 to 165.5 in 2020 (incidence rate ratio 1.016 per month, 95%CI:1.016–1.017, *p* < 0.001), and was not associated with COVID-19 (*p* = 0.96).

**Conclusions:**

In our setting, AMR-BSI incidence and etiology were similar between COVID-19 and non-COVID-19 cases. Incidence rates of hospital-origin AMR-BSI increased in 2020, which was likely due to increased blood culture utilization. We recommend increasing blood culture utilization and generating AMR surveillance reports in LMICs to inform local health care providers and policy makers.

**Supplementary Information:**

The online version contains supplementary material available at 10.1186/s13756-022-01114-x.

## Background

Antimicrobial-resistant (AMR) bacterial infections pose an emerging health problem globally, with a disproportionate impact in low and middle-income countries (LMICs) [1, 2]. The COVID-19 pandemic has potentially escalated this problem due to increased use of antibiotics in patients hospitalized with COVID-19 [3, 4].

Microbiology laboratories with blood culture facility hold a critical function of diagnosing the bacterial cause of infection and monitoring the AMR situation. The Surviving Sepsis Campaign International Guidelines recommend performing blood culture before starting antimicrobial therapy in patients presenting with sepsis [5]. Blood culture can be used to identify pathogenic organisms causing either community or hospital-acquired bloodstream infections (BSI); hence, blood culture results can guide definitive antimicrobial choices for each individual patient. In addition, cumulative antibiogram reports can be used to monitor the epidemiology of AMR infections and guide empirical antimicrobial choices to population [6].

There is a paucity of systematic surveillance networks evaluating blood culture utilization and burden of AMR infections in LMICs, including Indonesia. Indonesia is a lower-middle-income country in Southeast Asia with the world’s fourth largest population. A range of complex factors, e.g. limited laboratory infrastructure and limited specialized health care practitioners, lack of regulations on antimicrobial use and high burden of infectious diseases have hampered the implementation of the Indonesian National Action Plan for AMR [7–9]. Indonesia first reported AMR surveillance key indicators to the World Health Organization (WHO) Global Antimicrobial Resistance Surveillance System (GLASS) in 2021 [10]. The blood culture utilization in Indonesia is low (9% patients sampled for blood cultures out of all inpatients in Makassar versus 21% in Thailand in 2015) [11], which could lead to an underestimation of incidence rates and an overestimation of the proportion of AMR infection [12]. Thus, it is crucial to evaluate blood culture utilization rate together with the trend of AMR infections, particularly in LMICs [12].

Indonesia has been highly impacted by the COVID-19 pandemic. Following the first two confirmed cases of SARS-CoV-2 infection in Indonesia on 2 March 2020, there was a rapid increase with three pandemic waves of COVID-19 patients reaching 5.8 million confirmed cases and 1506,000 deaths countrywide at March 2022 [13]. Here, we evaluate blood culture utilization and epidemiology of AMR bloodstream infections in the Indonesian national referral hospital before and during the COVID-19 pandemic.

## Methods

### Study design, setting and population

We conducted a retrospective hospital-wide surveillance study by using routine data of all patients hospitalized at Cipto Mangunkusumo Hospital, the Indonesian national referral hospital, Jakarta, Indonesia, from 1 January 2019 to 31 December 2020. In response to increase number of COVID-19 cases in Indonesia, the hospital has expanded its capacity from 1,000 beds in 2019 to 1,125 beds in 2020, allocating 238 beds for COVID-19 cases and 887 beds for non-COVID-19 cases.

### Data collection

At the hospital, blood culture collection was determined by attending physicians based on the national standard practice [14]. Blood cultures were routinely performed at the microbiology laboratory of the Department of Clinical Pathology (International Organization for Standardization [ISO] 15,189, ISO 17205 and Joint Committee International accredited). A BacT/ALERT 3D automated microbial detection system machine expanded with additional incubator module (bioMerieux, Inc. Durham, USA) which can incubate up to 360 bottles was used. Isolated bacteria were identified using conventional bacterial identification methods and Vitek®2 (bioMerieux, Inc. Durham, USA). Antimicrobial susceptibility testing (AST) was performed using the Kirby-Bauer disc diffusion method according to Clinical and Laboratory Standards Institute guidelines [15].

Blood culture data were obtained through the Hospital Information System Management including the medical record number (MRN), admission date, specimen type, specimen date, culture and AST result. Hospital admission data were collected from the routine in-patient electronic records, and included MRN, admission date, discharge date and healthcare reimbursement program.

### Definitions

The blood culture utilization rate was defined as the ratio of the number of blood cultures per 1,000 patient-days [12]. Blood culture contamination was defined as the isolation of one or more common commensal organisms listed on National Healthcare Safety Network the Centers for Disease Control and Prevention list 2022 in only one set of blood culture or one of a series of two or more blood culture [16]. The blood culture contamination rate is defined as the ratio of the number of blood culture contamination per number of total blood cultures [17].

We used the definitions of infection origin as proposed by WHO GLASS. Community-origin (or hospital-origin) BSI was defined for patients in the hospital less (or more) than the first two calendar days of admission when the first blood specimen culture positive for a pathogen were taken, with calendar day one equal to the day of admission. For deduplication purposes, only the first isolate per patient, per pathogen, per year period was included in the analyses [18].

Our target pathogens were 12 bacteria species in the WHO global priority pathogens list; including carbapenem-resistant *Acinetobacter* spp*.* (CRACI), carbapenem-resistant *Pseudomonas aeruginosa* (CRPA), carbapenem-resistant or 3^rd^ generation cephalosporin-resistant *Klebsiella pneumoniae* (CRKP or 3GCRKP), carbapenem-resistant or 3^rd^ generation cephalosporin-resistant *Escherichia coli* (CREC or 3GCREC), vancomycin-resistant *Enterococcus faecium*, methicillin-resistant *Staphylococcus aureus* (MRSA), *Helicobacter pylori*, clarithromycin, fluoroquinolone-resistant *Campylobacter*, fluoroquinolone-resistant *Salmonella* spp, 3^rd^ generation cephalosporin-resistant or fluoroquinolone-resistant *Neisseria gonorrhoeae*, penicillin-non-susceptible *Streptococcus pneumoniae*, ampicillin-resistant *Haemophilus influenzae*, fluoroquinolone-resistant *Shigella* spp. [19].

The proportion of resistance was defined as the ratio of the number of patients having a positive blood culture for a WHO global priority resistant pathogen per the total number of patients having a positive blood culture for the given pathogen [18]. The incidence rate of community-origin AMR BSI is defined as the ratio of the number of patients with community-origin AMR BSI per 1,000 admissions. The incidence rate of hospital-origin AMR BSI is defined as the ratio of the number of patients with hospital-origin AMR BSI for each pathogen and antibiotic per 100,000 bed-days at risk of hospital-acquired infection. Moreover, as proposed by the WHO GLASS [18], we also estimated the incidence rate of AMR BSI per 100,000 tested patients as described previously [12].

A patient was classified as a COVID-19 case if their SARS-CoV-2 RT-PCR result was positive at any point during the admission period. We identified PCR-positive COVID-19 cases using the data of the healthcare imbursement program (Indonesian Case Based Groups [INA-CBG]) code of B34.2. which will cover confirmed COVID-19 patient expanses until cure). The year 2019 and 2020 was regarded as before and during the COVID-19 pandemic, respectively.

### Ethics

The study was approved by the Faculty of Medicine Universitas Indonesia Ethics Committee (KET-115/UN2.F1/ETIK/PPM.00.02/2021) and Oxford Tropical Research Ethics Committee (Reference: 503-22). The requirement for patient consent was waived as this was a secondary analysis of anonymised routine surveillance data. Permission was obtained from the hospital’s Innovation and Intellectual Property Directorate to use the routine hospital database for this study.

### Data analysis

Pearson’s chi-squared test and Fisher’s Exact test were used to compare categorical variables between groups. Kruskal Wallis test was used to compare continuous variables between groups.

We compared the blood culture utilization rate, contamination rate, isolated pathogens, and proportions and incidence rates of AMR BSI between COVID-19 and non-COVID-19 cases and between patients admitted in 2019 and 2020. Poisson regression models were used to assess changes in rate over time. All data analyses were performed using the STATA version 15.1 (StataCorp, College Station, TX, USA). We visualized figures with GraphPad Prism version 8.3.0 (La Jolla, California, USA). We also generated an overall AMR surveillance report using “AutoMated tool for Antimicrobial resistance Surveillance System (AMASS)” [20].

## Results

### Baseline characteristics

**Fig. 1:**
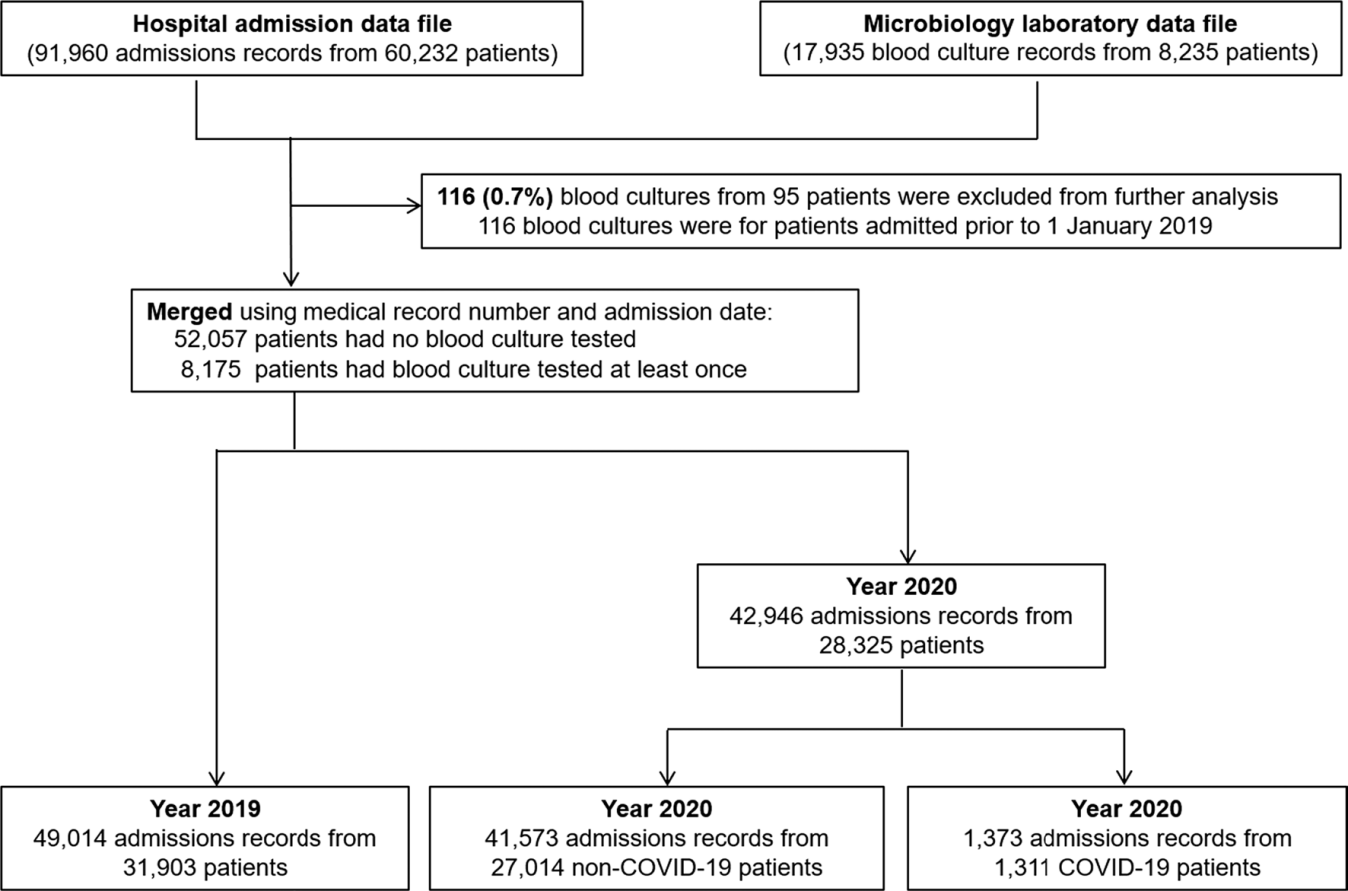
Title: Flow diagram

**Fig. 2:**
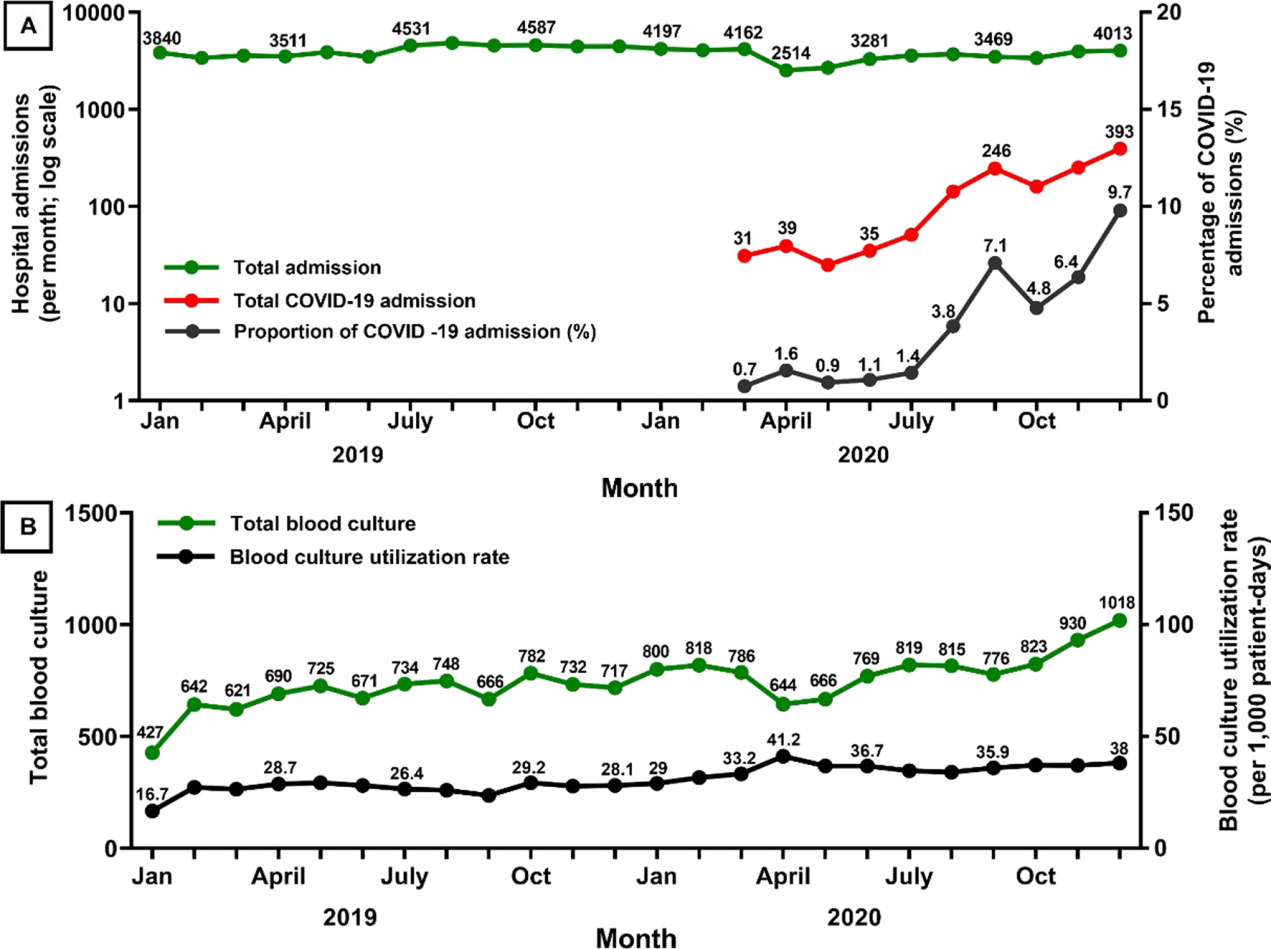
Numbers of patient admissions (**A**) and blood cultures (**B**) among inpatients from 2019 to 2020

Of 91,960 admissions (from 60,228 patients) admitted during the study period, 1,373 (from 1,311 patients) were COVID-19 cases (Fig. 1 and Table 1). In 2019, prior to the COVID-19 pandemic, the number of hospital admissions per month was relatively stable with a mean of 4,085 (range 3,374–4,818; Fig. 2A). At the start of the COVID-19 pandemic, the number of hospital admissions per month decreased sharply from 4,162 in March 2020 to 2,514 in April 2020 (39% decrease). The proportion of COVID-19 admissions per total admissions increased from 0.7% (31 admissions) in March 2020 and reached the highest to 9.7% (393 admissions) in December 2020. Overall, the total numbers of admissions per year was higher in 2019 at 49,014 (of 31,903 patients) than in 2020 at 42,946 (of 28,325 patients) (12% difference; Table 1).

**Table 1:** Baseline characteristics by year and by COVID-19 status

Parameters	Year 2019	Year 2020	*P* values	Non-COVID-19 cases	COVID-19 cases*	*P* values
Total number of admissions	49,014	42,946	–	90,587	1,373	–
Total number of inpatients (de-duplicated)	31,903	28,325	–	58,917	1,311	–
Number of patient-days	308,926	274,322	–	571,707	11,541	–
Number of blood culture specimens received	8,155	9,664	–	17,286	533	–
Number of blood culture positive for any organism	1,589	2,025	–	3,513	101	–
Blood culture positivity rate	19.5%	20.9%	0.02	20.3%	18.9%	0.44
Number of blood culture positive for commensal bacteria**	438	570	–	977	32	–
Blood culture contamination rate**	5.4%	5.9%	0.13	5.7%	6.0%	0.73
Number of patients sampled for blood cultures (de-duplicated)	4,026	4,501	–	7,973	348	–
Prevalence of patients sampled for blood cultures among all inpatients	12.6%	15.9%	< 0.001	13.5%	26.5%	< 0.001
Average number of blood culture specimens sampled per admission	1.6	1.7	–	1.7	1.5	–
Total number of admissions that had at least two blood culture specimens sampled (%)	2,472 (5.0%)	2,889 (6.7%)	< 0.001	5,174 (5.7%)	187 (13.6%)	< 0.001
Median duration between the first and second blood culture specimen (days, IQR)***	5 (3–9)	5 (3–8)	0.52	5 (3–8)	4 (2–7)	0.57
Blood culture utilization rate (per 1,000 patient-days)	26.4	35.1	< 0.001	30.2	43.5	< 0.001
*Blood culture utilization for community-origin BSI*						
Prevalence of blood culture specimens being collected within the first 2 calendar days of hospital admission	34.3% (2,801/8,155)	31.9% (3,087/9,664)	0.01	32.7% (5,650/17,286)	45.0% (240/533)	< 0.001
Number of patients tested for community-origin BSI (de-duplicated) ****	1,747	2,481	–	4,458	176	–
*Blood culture utilization for hospital-origin BSI*						
Prevalence of blood culture specimens being collected after the first 2 calendar days of hospital admission	65.7% (5,354/8,155)	68.1% (6,577/9,664)	0.01	67.3% (11,636/17,286)	55.0% (293/533)	< 0.001
Number of patients tested for hospital-origin BSI (de-duplicated) ****	2,556	2,385	–	4,175`	176	–

*BSI* Bloodstream infections

*All COVID-19 cases were in 2020

**Commensal bacteria included coagulase-negative *Staphylococci, viridans group Streptococci, Propionibacterium acnes, Corynebacterium* spp., and *Bacillus* spp.

***Among admissions that had at least two blood culture specimens sampled

****Patients tested for community-origin BSI were defined as patients with the first blood culture performed within the first two calendar days of admissions during the reporting period. Patients tested for hospital-origin BSI were defined as patients with the first blood culture performed after the first two calendar days of admissions during the reporting period

### Blood culture utilization

Of 60,228 patients, 8,175 had at least one blood culture taken (total 17,819 blood cultures). Total patient-days during the study period were 583,248, giving a blood culture utilization rate of 30.6 per 1,000 patient-days. 2,735 patients had at least two blood cultures sampled within a single admission, and the median duration between the first and second blood culture was 5 calendar days (IQR 3–8 calendar days).

The blood culture utilization rate showed an increasing trend over 2019, before the COVID-19 pandemic, from 16.7 per 1,000 patient-days in January 2019 to 28.1 in December 2019 (utilization rate ratio [URR] 1.02 per month; 95%CI 1.01–1.02, *p* < 0.001, Fig. 2B). This increasing trend continued throughout 2020 (URR 1.02 per month; 95%CI 1.01–1.02, *p* < 0.001).

The blood culture utilization rate was higher among COVID-19 cases compared to non-COVID-19 cases (43.5 vs. 30.2 per 1,000 patient-days; *p* < 0.001; Table 1). In a multivariable Poisson regression model, the blood culture utilization rate was independently associated with time (adjusted URR [aURR]: 1.02 per month, 95% CI 1.01–1.02, *p* < 0.001) and COVID-19 cases (aURR 1.19, 95% CI 1.09–1.30, *p* < 0.001).

### Isolated organisms

Of 17,819 blood cultures, 1,008 were positive for commensal bacteria, giving a blood culture contamination rate of 5.6% during the study period. Of 8,175 patients who had at least one blood culture taken, 1,895 (23.1%) had at least one blood culture positive for one or more pathogenic organisms.

Among patients with BSI, 1,342 (70.8%) were Gram-negative bacteria, 296 (15.6%) were Gram-positive bacteria, 205 (10.8%) were fungi and 52 (2.8%) were polymicrobial infections (Table 2 and Additional file 1: Table S1). The most common pathogens identified were *K. pneumoniae* (23.3%; n = 442), *Acinetobacter* spp. (13.9%; n = 263), *E. coli* (13.1%; n = 249), *S. aureus* (11.6%; n = 219) and *P. aeruginosa* (8.6%; n = 163).

**Table 2:** Pathogenic organisms isolated from 1,895 patients with bloodstream infections between 2019 and 2020*

Pathogens	Year 2019 (N = 828)	Year 2020 (N = 1067)	*P* values	Non COVID-19 cases (N = 1838)	COVID-19 cases** (N = 57)	*P* values
*Gram negative bacteria*						
*Escherichia coli*	115 (13.9%)	115 (10.7%)	0.04	221 (12%)	9 (15.8%)	0.39
*Klebsiella pneumonia*	201 (24.3%)	207 (19.4%)	0.01	398 (21.7%)	10(17.5%)	0.45
*Klebsiella* spp	16 (1.9%)	18 (1.7%)	0.69	32 (1.7%)	2 (3.5%)	0.32
*Proteus* spp	7 (0.9%)	12 (1.1%)	0.54	19 (1%)	0 (0%)	0.44
Salmonella spp	21 (2.5%)	12 (1.1%)	0.02	33 (1.8%)	0 (0%)	0.62
*Salmonella enterica*	3 (0.4%)	1 (0.1%)	0.32	4 (0.2%)	0 (0%)	> 0.99
*S. enterica* serovar typhi	2 (0.2%)	4 (0.4%)	0.70	5 (0.3%)	1 (1.8%)	0.05
*Shigella* spp	1 (0.1%)	0 (0%)	0.43	1 (0.1%)	0 (0%)	> 0.99
*Pseudomonas* *aeruginosa*	59 (7.1%)	98 (9.2%)	0.10	152 (8.3%)	5 (8.8%)	0.89
*Pseudomonas* spp	1 (0.1%)	3 (0.3%)	0.63	4 (0.2%)	0 (0%)	> 0.99
*Acinetobacter* spp	102 (12.3%)	151 (14.1%)	0.24	245 (13.3%)	8 (14%)	0.87
*Aeromonas* spp	5 (0.6%)	5 (0.5%)	0.75	10 (0.5%)	0 (0%)	> 0.99
*Burkholderia cepacia*	6 (0.8%)	7 (0.7%)	0.85	13 (0.7%)	0 (0%)	> 0.99
*Citrobacter* spp	4 (0.5%)	3 (0.3%)	0.70	7 (0.4%)	0 (0%)	> 0.99
*Serratia* spp	11 (1.3%)	6 (0.6%)	0.07	17 (0.9%)	0 (0%)	> 0.99
Other Gram-negative bacteria	63 (7.6%)	83 (7.7%)	0.89	143 (7.8%)	3 (5.3%)	0.48
*Gram positive bacteria*						
*Staphylococcus aureus*	88 (10.6%)	128 (12%)	0.35	213 (11.6%)	3 (5.3%)	0.13
*Streptococcus pneumoniae*	2 (0.2%)	1 (0.1%)	0.58	2 (0.1%)	1 (1.8%)	0.08
*Streptococcus pyogenes*	2 (0.2%)	(0.2%)	> 0.99	4 (0.2%)	0 (0%)	> 0.99
*Enterococcus faecium*	6 (0.8%)	5 (0.5%)	0.54	11 (0.6%)	0 (0%)	> 0.99
*Enterococcus faecalis*	22 (2.7%)	39 (3.6%)	0.22	55 (3.0%)	6 (10.4%)	0.01
*Lactococcus garvieae*	0 (0%)	1 (0.1%)	> 0.99	1 (0.1%)	0 (0%)	> 0.99
*Fungi*						
*Candida albicans*	7 (0.9%)	30 (2.8%)	0.01	34 (1.8%)	3 (5.3%)	0.67
Non-albicans *Candida* spp	53 (6.4%)	110 (10.3%)	0.01	157 (8.5%)	6 (10.5%)	0.59
*Cryptococcus* spp	2 (0.2%)	0 (0%)	0.19	2 (0.1%)	0 (0%)	> 0.99
Other fungi	0 (0%)	3 (0.3%)	0.26	3 (0.3%)	0 (0%)	> 0.99
Polymicrobial infections***	29 (3.5%)	23 (2.2%)	0.07	52 (2.8%)	0 (0%)	0.40

*BSI* Bloodstream infections

*Only the first pathogenic isolate per patient during the study period was included

**All COVID-19 cases were in 2020

***Three most common polymicrobial infections were *Escherichia coli* + *Klebsiella pneumoniae* (10 patients), *Klebsiella pneumoniae* + Other Gram-negative bacteria (10 patients), *Acinetobacter* spp. + *Klebsiella pneumoniae* (7 patients). Polymicrobial infections are described in Addition file 1: Table S2

The proportion of isolated pathogens among BSI patients was moderately different between 2019 and 2020 (Table 2). The isolated pathogens were not different between COVID-19 and non-COVID-19 cases (all *p* > 0.05), except that the proportion of *Enterococcus faecalis* was lower in non-COVID-19 than COVID-19 cases (3.0% vs. 10.4% *p* = 0.01).

The most common pathogens identified as the cause of community-origin BSI was *E. coli* (20%; n = 103/515), followed by *S. aureus* (16.9%; n = 87/515) and *K. pneumoniae* (11.1%; n = 57/515), while the most common pathogens identified as the cause of hospital-origin BSI was *K. pneumoniae* (25.4%; n = 351/1,380), followed by *Acinetobacter* spp. (14%; n = 193/1,380) and non-albicans *Candida* (10.7%; n = 16/1,380) (Additional file 1: Table S1 and S2).

### Proportion of AMR BSI

Of 442 patients with BSI caused by *K. pneumoniae*, 371 (83.9%) and 160 (36.2%) were caused by 3GCRKP and CRKP, respectively (Table 3). Of 249 patients with BSI caused by *E. coli*, 187 (76.1%) and 34 (13.6%) were caused by 3GCREC and CREC, respectively. All CREC and CRKP were also resistant to 3GC. The proportion of CRACI was 46.8% (123/263).

**Table 3:** Proportion of WHO global priority AMR pathogens causing bloodstream infections

Priority AMR pathogens*	Year 2019	Year 2020	*P* values	Non COVID-19 cases	COVID-19 cases**	*P* values
Carbapenem resistant *Acinetobacter* spp.	46% (48/105)	48.7% (77/158)	0.56	48.2% (123/255)	25% (2/8)	0.29
Carbapenem resistant *P. aeruginosa*	27% (17/64)	24.2% (24/99)	0.74	26% (41/158)	0% (0/5)	0.33
Carbapenem resistant *** *K. pneumoniae*	34% (75/218)	38% (85/224)	0.44	35.9% (155/432)	50% (5/10)	0.51
3^rd^ Cephalosporin resistant *** *K. pneumoniae*	85.3% (186/218)	82.5% (185/224)	0.43	83.8% (362/432)	90% (9/10)	> 0.99
Carbapenem resistant *** *E*. *coli*	16.3% (21/129)	10.8% (13/120)	0.21	13% (31/240)	34% (3/9)	0.11
3^rd^ Cephalosporin resistant *** *E*. *coli*	76% (98/129)	74.2% (89/120)	0.74	75% (180/240)	77.8% (7/9)	> 0.99
Vancomycin resistant *E*. *faecium*	0% (0/7)	20% (1/5)	0.42	8.3% (1/12)	0% (0/0)	
Methicillin resistant *S*. *aureus*	3.4% (3/88)	9.2% (12/131)	0.11	6.9% (15/216)	0% (0/3)	> 0.99
Fluoroquinolone resistant *Salmonella* spp.	17.9% (5/28)	5.9% (1/17)	0.38	13.3% (6/45)	0% (0/0)	> 0.99
Fluoroquinolone resistant *Shigella* spp.	100% (1/1)	0% (0/0)		100% (1/1)	0% (0/0)	
Penicillin resistant S. *pneumoniae*	50% (1/2)	0% (0/1)	> 0.99	50% (1/2)	0% (0/1)	> 0.99
Overall****	55.9% (359/642)	51.5% (389/755)	0.10	53.6% (730/1361)	50% (18/36)	0.67

*CO* Community-origin, *HO* Hospital-origin

CO and HO were defined as proposed by WHO GLASS [18])

*Only the first pathogenic isolate per patient during the study period was included

**All COVID-19 cases were in 2020

***All carbapenem-resistant *E. coli* and *K. pneumoniae* were also resistant to 3^rd^ cephalosporin cephalosporin

****Among patients with blood culture positive for *Acinetobacter* spp., *P. aeruginosa*, *K. pneumoniae, E*. *coli, E*. *faecium, S*. *aureus, Salmonella* spp, *Shigella* spp or S. *pneumoniae*

The proportion of AMR for each priority pathogen was not different between 2019 and 2020 (all *p* > 0.10), and between COVID-19 and non-COVID-19 cases (all *p* > 0.10; Table 3). However, the proportion of AMR for each priority pathogen were different between community-origin BSI and hospital-origin BSI (Additional file 1: Table S3). For example, the proportion of 3GCRKP (61.5% vs. 87.8%, *p* < 0.001), 3GCREC (67.8% vs. 77.6%, *p* = 0.02) and CRACI (16.4% vs. 56.9%, *p* < 0.001) were lower among community-origin BSI compared to those of hospital-origin BSI. Additional file 1: Table S4 provides additional details on proportion of AMR stratified by infection origin (community-origin vs. hospital-origin) and COVID-19 status. The overall AMR surveillance report is provided in Additional file 2.

### Incidence rates of AMR BSI

The incidence rate of community-origin AMR BSI per 1,000 admissions was not significantly different between year 2019 and 2020 (1.6 to 1.6 per 1,000 admissions, *p* = 0.97; Table 4), while the incidence rate of hospital-origin AMR BSI per 100,000 patient-days in 2020 (165.5 per 100,000 patient-days at risk) was higher than 2019 (130.1 per 100,000 patient-days at risk) (*p* = 0.003). No specific outbreaks of AMR BSI were observed during the study period.

**Table 4:** Incidence rate of WHO global priority AMR pathogens causing bloodstream infections

	Year 2019	Year 2020	*P* values	Non COVID-19 cases	COVID-19 cases**	*P* values
*Incidence rate of community-origin BSI caused by WHO global priority AMR pathogens*						
per 1,000 admissions	1.6 (76/49,014)	1.6 (67/42,946)	0.97	1.6 (141/90,587)	1.5 (2/1,373)	> 0.99
per 100,000 patients tested for community-origin BSI*	4,350.3 (76/1,747)	2,700.5 (67/2,481)	0.004	3,162.8 (141/4,458)	1,136.3 (2/176)	0.11
*Incidence rate of hospital-origin BSI caused by WHO global priority AMR pathogens*						
per 1,000 admissions at risk of hospital-origin BSI*	8.5 (283/33,226)	10.9 (322/29,510)	0.003	9.5 (589/61,758)	14.6 (16/1,089)	0.10
per 100,000 patient-days at risk of hospital-origin BSI*	130.1 (283/217,398)	165.5 (322/194,486)	0.003	146.9 (589/400,750)	143.7 (16/11,134)	0.96
per 100,000 patients tested for hospital-origin BSI*	11,071.9 (283/2,556)	13,501.1 (322/2,385)	0.01	14,107.7 (589/4,175)	9,090.9 (16/176)	0.07

*BSI* Bloodstream infections

*Patients tested for community-origin BSI were defined as patients with the first blood culture performed within the first two calendar days of admissions during the reporting period. Patients were considered at risk of hospital-origin BSI after they stayed in the hospital for more than 2 days. Patients tested for hospital-origin BSI were defined as patients with the first blood culture performed after the first two calendar days of admissions during the reporting period

**All COVID-19 cases were in 2020

The incidence rate of community-origin AMR BSI per 1,000 admissions was not different between COVID-19 and non-COVID-19 cases during 2019 and 2020 (1.5 vs. 1.6, *p* > 0.99). The incidence rate of hospital-origin AMR BSI per 100,000 patient-days at risk was also not different between COVID-19 and non-COVID-19 cases (143.7 vs. 146.9, *p* = 0.96).

We observed that the incidence rate of community-origin AMR BSI per 100,000 tested patients was higher in 2019 compared with 2020 (4350.3 vs. 2,700.5 per 100,000 tested patients, *p* = 0.004), while incidence rate of hospital-origin AMR BSI per 100,000 tested patients was lower in 2019 (11,071.9 vs. 13,501.1 per 100,000 tested patients, *p* = 0.01; Table 4). We found that incidence rate of community-origin AMR BSI and of hospital-origin AMR BSI per 100,000 tested patients was not significantly different between non-COVID-19 and COVID-19 cases (*p* = 0.11 and *p* = 0.07, respectively).

## Discussion

This study illustrates that the use of readily available electronic hospital databases could provide robust and useful information on blood culture utilization and burden of AMR infections before and during the COVID-19 pandemic in LMICs. Although several reports have recently described an increase in AMR infections among COVID-19 cases [21, 22], we did not observe a difference of AMR BSI between COVID-19 and non-COVID-19 cases during the same time period in our setting. Strikingly, our study showed that the blood culture utilization rate had been increasing at the hospital before the COVID-19 pandemic (in 2019) and during the pandemic (in 2020), and, furthermore, that it was higher in COVID-19 cases than non-COVID-19 cases.

We did not observe a clear difference in the proportion and incidence rate of AMR infections between COVID-19 and non-COVID-19 cases, which is consistent with a study from Singapore [23]. Improved infection prevention control in hospitals and communities, and reduced mobilization in community could hypothetically explain this finding [24]. Nonetheless, studies from China [25], India [22], Italy [26], Taiwan [21], reported an increase in the proportion or incidence rates of AMR infections in COVID-19 patients. Multiple possible reasons for an increase include the high antibiotic use, predominance of severe COVID-19 patients in intensive care unit (ICU) with multiple predispositions towards AMR infections and protracted hospital stay, overcrowding of patients, and limited guideline adherence [27–31]. Therefore, appropriate antimicrobial prescribing, accurate diagnosis and appropriate infection prevention control are crucial for both COVID-19 and non-COVID-19 patients.

The observed increase in the incidence rate of hospital-origin AMR BSI during the COVID-19 pandemic is most likely due to the increase in blood culture utilization rate. A simulation study showed that observed incidence rate of AMR BSI (per 100,000 patient-days) could considerably increase if a hospital improves their blood culture utilization rate even if there are no changes in true susceptibility profiles of pathogenic organisms and in true infection rates in that environment over time [12]. We did not observe changes in proportion of AMR BSI and specific outbreaks of AMR infections during the study period, as was noticed by Hospital Infection Prevention Control Committee.

The observed increase in blood culture utilization rate before and during the COVID-19 pandemic could be due to several reasons. Before the COVID-19 pandemic, a new national clinical practice guideline on sepsis was launched including the recommendation to take blood cultures prior to start of empirical antibiotic therapy [14]. Adoption of this guideline in the hospital is likely to have contributed to the increase observed. Nonetheless, the blood culture utilization rate in non-COVID-19 cases was still lower than those reported in Thailand and many other high-income countries (e.g. 307.7, 86.5, 65.4 per 1,000 patient-days in United States, France and United Kingdom, respectively) [11, 32, 33]. Direct comparison of the rate with other LMICs could not be performed due to limited existing publication [34]. The low culture rate could be explained by lack of physician awareness of the sepsis guidelines, misperceptions that blood culture will add health care cost, among other factors [35]. Previous studies have reported contrasting findings on blood culture utilization in COVID-19 patients [22, 36, 37]. The increase in blood culture utilization among COVID-19 cases in our hospital is probably because the national referral hospital manages mostly COVID-19 patients with comorbidities and severe conditions. Given these are very sick people empirical antibiotic treatment is commonly recommended [38]. However, a recent meta-analysis has concluded that antibiotics are heavily overused in COVID-19 cases [39].

Local reporting on the hospital AMR epidemiology allows us to understand the local situation and support local actions. The top three pathogens causing BSIs are similar with findings in other countries in the region [10, 40]. However, we did not observe higher rate of *S. aureus* co-infection in COVID-19 patients, contrary to several reports from past viral and COVID-19 pandemic worldwide [41]. In addition, our analysis shows BSI cases with *Salmonella enterica* serovar Typhi*, Shigella* spp.*,* and *Burkholderia pseudomallei*. Those pathogens are the cause of typhoid, shigellosis and melioidosis, respectively, and are notifiable pathogens in many countries [42, 43]. This information can support the decision making of the Ministry of Health in Indonesia, where a system of notifiable pathogens is not officially established. Our study also found that 8.6% of hospital-acquired BSI was caused by non-albicans *Candida spp*. There is limited information of fungal infections, particularly of non-albicans *Candida* infections, as the cause of BSI in LMICs [44–47] The relatively high proportion of fungemia in our data, compared with 1.1% in Thailand [46], could be due the complex, immunocompromised patient populations, with common invasive procedures and high antibiotic use, all of which are risk factors of invasive candidiasis [31, 48]. Available data worldwide suggest increasing incidence of fungemia caused by non-albicans *Candida* species [44, 49, 50] together with increasing resistance. We reported our findings to the hospital Infection and Antimicrobial Resistance Control Committee, and these are used to support local guidelines for the prevention and treatment of hospital acquired invasive fungal infections [50, 51].

Our study has some limitations. First, we could not determine whether a blood culture was taken before or after failure of empirical treatment as there was a low adherence to take blood culture prior to antibiotic treatment. Some patients may also be treated with parenteral antibiotics without blood culture taken. Implementation of case-based instead of laboratory-based surveillances could improve data representativeness in the future. Second, our AMR surveillance reports should not be used to guide empirical antibiotics without careful consideration. Hospitals in LMICs with a low blood culture utilization rate should use AMR surveillance reports stratified by exposure to an empirical antibiotic at the study hospital to guide choice of first-line empiric antimicrobial therapy rather than the total antibiogram [12]. Third, although large, our study may lack of power to observe a difference in AMR BSI between COVID-19 and non-COVID-19 cases. Fourth, we use calendar days of admission as a surrogate for defining origin of infection and data of patient transfers are not available. Therefore, a proportion of community-origin BSIs reported could be hospital-origin BSIs transferring from other hospitals. Lastly, the findings may not be generalizable to all other hospitals or the country at large.

## Conclusions

In our setting, AMR BSIs were not different between COVID-19 and non-COVID-19 cases. Increased incidence rates of hospital-origin AMR infections observed in 2020 could be due to increasing blood culture utilization rate. Systematic, representative AMR data are required to better estimate the extent of the problem, and adequately inform antibiotic guidelines and stewardship programs. We recommend hospitals in LMICs to increase blood culture utilization and generate annual AMR surveillance reports together with parameters representing blood culture utilization.

## Supplementary Information

**Additional file1**: Supplementary Table 1: Polymicrobial Pathogenic organisms isolated from 52 patients with bloodstream infections at Cipto Mangunkusumo National Hospital, Indonesia, between 2019 and 2020. Supplementary Table 2: Pathogenic organisms isolated from 1,895 patients with bloodstream infections at Cipto Mangunkusumo National Hospital, Indonesia, between 2019 and 2020. Supplementary Table 3: Prevalence of WHO global priority AMR pathogens causing bloodstream infections stratified by infection origin. Supplementary Table 4. Proportion of WHO global priority AMR pathogens causing bloodstream infections stratified by infection origin and COVID-19 status

**Additional file2**: Antimicrobial Resistance (AMR) Surveillance Report. Antimicrobial Resistance (AMR) Surveillance Report of the study site generated by AMASS

## Data Availability

The datasets used and/or analyzed during the current study available from the corresponding author on reasonable request.

## Abbreviations

AMR: Antimicrobial-resistant
AST: Antimicrobial susceptibility testing
AMASS: AutoMated tool for Antimicrobial resistance Surveillance System
BSI: Bloodstream infections
CRACI: Carbapenem-resistant *Acinetobacter spp.*
CREC: Carbapenem-resistant *Escherichia coli*
CRKP: Carbapenem-resistant *Klebsiella* *pneumoniae*
CRPA: Carbapenem-resistant *Pseudomonas* *aeruginosa*
GLASS: Global antimicrobial resistance surveillance system
INA-CBG: Indonesian case based groups
ICU: Intensive care unit
LMIC: Low and middle-income country
MRN: Medical record number
MRSA: Methicillin-resistant *Staphylococcus aureus*
3GCREC: 3^rd^ generation cephalosporin-resistant *Escherichia coli*
3GCRKP: 3^rd^ generation cephalosporin-resistant *Klebsiella pneumoniae*
WHO: World Health Organization
