# Supplementary material for: Blood culture utilization and epidemiology of antimicrobial-resistant bloodstream infections before and during the COVID-19 pandemic in the Indonesian national referral hospital
Source: Antimicrob Resist Infect Control. 2022 May 19;11:73. doi: 10.1186/s13756-022-01114-x (PMC9117993; doi:10.1186/s13756-022-01114-x)
Supplement: Supplementary file 1 — Additional file1: Supplementary Table 1: Polymicrobial Pathogenic organisms isolated from 52 patients with bloodstream infections at Cipto Mangunkusumo National Hospital, Indonesia, between 2019 and 2020. Supplementary Table 2: Pathogenic organisms isolated from 1,895 patients with bloodstream infections at Cipto Mangunkusumo National Hospital, Indonesia, between 2019 and 2020. Supplementary Table 3: Prevalence of WHO global priority AMR pathogens causing bloodstream infections stratified by infection origin. Supplementary Table 4. Proportion of WHO global priority AMR pathogens causing bloodstream infections stratified by infection origin and COVID-19 status [file 13756_2022_1114_MOESM1_ESM.docx]

**Supplementary Table 1**. Polymicrobial Pathogenic organisms isolated from 52 patients with bloodstream infections at Cipto Mangunkusumo National Hospital, Indonesia, between 2019 and 2020

| **Pathogens** | **Total* (N=52)** | **Year 2019**  **(N=29)** | **Year 2020**  **(N=23)** | **Community-origin BSI**  **(N=14)** | **Hospital-origin BSI**  **(N=38)** |
| --- | --- | --- | --- | --- | --- |
| *Acinetobacter* spp. + *Klebsiella pneumoniae* | 7 | 2 | 5 | 1 | 6 |
| *Acinetobacter* spp. + *Pseudomonas aeruginosa* | 2 | 1 | 1 | 0 | 2 |
| *Acinetobacter* spp. + *Staphylococcus aureus* | 1 | 0 | 1 | 0 | 1 |
| *Citrobacter* spp. + *Escherichia coli* | 1 | 0 | 1 | 1 | 0 |
| *Escherichia coli + Klebsiella pneumoniae* | 10 | 7 | 3 | 4 | 6 |
| *Escherichia coli* + *Klebsiella* spp. | 2 | 2 | 0 | 2 | 0 |
| *Escherichia coli* + *Proteus* spp. | 2 | 1 | 1 | 2 | 0 |
| *Escherichia coli* + *Pseudomonas aeruginosa* | 1 | 1 | 0 | 0 | 1 |
| *Escherichia coli* + *Salmonella* spp. | 1 | 1 | 0 | 1 | 0 |
| *Escherichia coli* + Other Gram-negative bacteria | 2 | 2 | 0 | 0 | 2 |
| *Enterococcus faecium* + *Klebsiella pneumoniae* | 1 | 1 | 0 | 0 | 1 |
| *Enterococcus faecalis* + *Staphylococcus aureus* | 2 | 0 | 2 | 0 | 2 |
| *Enterococcus faecalis* + Other Gram-negative bacteria | 1 | 1 | 0 | 0 | 1 |
| *Klebsiella pneumoniae* + *Pseudomonas aeruginosa* | 2 | 2 | 0 | 0 | 2 |
| *Klebsiella pneumoniae* + *Proteus* spp. | 2 | 1 | 1 | 1 | 1 |
| *Klebsiella pneumoniae* + *Serratia* spp. | 1 | 1 | 0 | 0 | 1 |
| *Klebsiella pneumoniae* + Non-albicans *Candida* | 1 | 1 | 0 | 0 | 1 |
| *Klebsiella pneumoniae* + Other Gram-negative bacteria | 10 | 2 | 8 | 2 | 8 |
| *Klebsiella* spp. + *Salmonella enterica* | 1 | 1 | 0 | 0 | 1 |
| *Klebsiella* spp. + Other Gram-negative bacteria | 1 | 1 | 0 | 0 | 1 |
| *Pseudomonas aeruginosa* + Other Gram-negative bacteria | 1 | 1 | 0 | 0 | 1 |

BSI = bloodstream infections ***** All patients with BSI caused by polymicrobial pathogenic organism were non-COVID-19 cases. Community-origin and hospital-origin BSI are defined as proposed by WHO GLASS.

**Supplementary Table 2**. Pathogenic organisms isolated from 1,895 patients with bloodstream infections at Cipto Mangunkusumo National Hospital, Indonesia, between 2019 and 2020 *

| **Pathogens** | **Community-origin BSI**  **(N=515)** | **Hospital-origin BSI**  **(N=1380)** | **P value** |
| --- | --- | --- | --- |
| **Gram negative bacteria** |  |  |  |
| *Escherichia coli* | 103 (20%) | 127 (9.2%) | <0.001 |
| *Klebsiella pneumonia* | 57 (11.1%) | 351 (25.4%) | <0.001 |
| *Klebsiella* spp. | 10 (1.9%) | 24 (1.7%) | 0.76 |
| *Proteus* spp. | 5 (1%) | 14 (1%) | 0.93 |
| *Salmonella* spp. | 18 (3.5%) | 15 (1.1%) | <0.001 |
| *Salmonella enterica* | 2 (0.4%) | 2 (0.1%) | 0.29 |
| *S. enterica serotype typhi* | 6 (1.2%) | 0 (0%) | <0.001 |
| *Shigella* spp. | 1 (0.2%) | 0 (0%) | 0.27 |
| *Pseudomonas aeruginosa* | 62 (12%) | 95 (6.9%) | <0.001 |
| *Pseudomonas* spp. | 1 (0.2%) | 3 (0.2%) | >0.99 |
| *Acinetobacter* spp. | 60 (11.7%) | 193 (14%) | 0.18 |
| *Aeromonas* spp. | 3 (0.6%) | 7 (0.5%) | 0.73 |
| *Burkholderia cepacian* | 4 (0.8%) | 9 (0.7%) | 0.75 |
| *Citrobacter* spp. | 0 (0%) | 7 (0.5%) | 0.20 |
| *Serratia* spp. | 2 (0.4%) | 15 (1.1%) | 0.39 |
| Other Gram-negative bacteria | 39 (7.6%) | 107 (7.8%) | 0.89 |
| **Gram positive bacteria** |  |  |  |
| *Staphylococcus aureus* | 87 (16.9%) | 129 (9.4%) | <0.001 |
| *Streptococcus pneumoniae* | 3 (0.6%) | 0 (0%) | 0.02 |
| *Streptococcus pyogenes* | 4 (0.8%) | 0 (0%) | 0.01 |
| *Enterococcus faecium* | 1 (0.2%) | 10 (0.7%) | 0.30 |
| *Enterococcus faecalis* | 15 (2.9%) | 46 (3.3%) | 0.64 |
| *Lactococcus garvieae* | 0 (0%) | 1 (0.1%) | >0.99 |
| **Fungi** |  |  |  |
| *Candida* *albicans* | 2 (0.4%) | 35 (2.5%) | 0.01 |
| Non-albicans *Candida* | 16 (3.1%) | 147 (10.7%) | <0.001 |
| *Cryptococcus* spp. | 0 (0%) | 2 (0.1%) | >0.99 |
| Other fungi | 0 (0%) | 3 (0.2%) | 0.56 |
| **Polymicrobial infections**** | 14 (2.7%) | 38 (2.8%) | 0.96 |

BSI = bloodstream infections * Only the first pathogenic isolate per patient during the study period was included. ** Three most common polymicrobial infections were *Escherichia coli + Klebsiella pneumoniae* (10 patients), *Klebsiella pneumoniae* + Other Gram-negative bacteria (10 patients), *Acinetobacter* sp + *Klebsiella pneumoniae* (7 patients). Polymicrobial infections was described in Supplementary Table 1.

**Supplementary Table 3.** Prevalence of WHO global priority AMR pathogens causing bloodstream infections stratified by infection origin

| **Priority AMR pathogens *** | **Community-origin** | **Hospital-origin** | **P value** |
| --- | --- | --- | --- |
| Carbapenem resistant *Acinetobacter* spp. | 16.4% (10/61) | 56.9% (115/202) | <0.001 |
| Carbapenem resistant  *P. aeruginosa* | 11.3%  (7/62) | 33.7% (34/101) | <0.001 |
| Carbapenem resistant ***  *K.* *pneumoniae* | 21.5% (14/65) | 38.7% (146/377) | 0.01 |
| 3^rd^ Cephalosporin resistant *** *K.* *pneumoniae* | 61.5% (40/65) | 87.8% (331/377) | <0.001 |
| Carbapenem resistant *** *E*. *coli* | 11.6% (13/112) | 15.3% (21/137) | 0.40 |
| 3^rd^ Cephalosporin resistant *** *E*. *coli* | 67.8% (76/112) | 77.6% (111/137) | 0.02 |
| Vancomycin resistant  *E*. *faecium* | 0%  (0/1) | 9.1% (1/11) | >0.99 |
| Methicillin resistant  *S*. *aureus* | 5.8%  (5/87) | 7.6% (10/132) | 0.60 |
| Fluoroquinolone resistant  *Salmonella* spp. | 11.1%  (3/27) | 16.7% (3/18) | 0.67 |
| Fluoroquinolone resistant  *Shigella* spp. | 100%  (1/1) | 0%  (0/0) |  |
| Penicillin resistant  S. *pneumoniae* | 33.3%  (1/3) | 0%  (0/0) |  |
| Overall**** | 34.1%  (143/419) | 61.8%  (605/978) | <0.001 |

CO = community-origin. HO = hospital-origin. CO and HO are defined as proposed by WHO GLASS.(WHO, 2020) * Only the first pathogenic isolate per patient during the study period was included. ****** All COVID-19 cases were in 2020. *** All carbapenem-resistant *E. coli* and *K. pneumoniae* were also resistant to 3^rd^ cephalosporin. **** Among patients with blood culture positive for *Acinetobacter* spp., *P. aeruginosa*, *K.* *pneumoniae, E*. *coli, E*. *faecium, S*. *aureus, Salmonella* spp, *Shigella* spp or S. *pneumoniae*

**Supplementary Table 4.** Proportion of WHO global priority AMR pathogens causing bloodstream infections stratified by infection origin and COVID-19 status

| **Priority AMR pathogens *** | **Community-origin** | | | **Hospital-origin** | | |
| --- | --- | --- | --- | --- | --- | --- |
|  | **Non-COVID-19 cases** | **COVID-19 cases** | **P values** | **Non-COVID-19 cases** | **COVID-19 cases** | **P values** |
| Carbapenem resistant *Acinetobacter* spp. | 17%  (10/59) | 0%  (0/2) | >0.99 | 57.6% (113/196) | 33.3%  (2/6) | 0.41 |
| Carbapenem resistant  *P. aeruginosa* | 12%  (7/58) | 0%  (0/4) | >0.99 | 43%  (43/100) | 0%  (0/1) | >0.99 |
| Carbapenem resistant ***  *K.* *pneumoniae* | 21,8%  (14/64) | 0%  (0/1) | >0.99 | 38%  (141/372) | 55.5%  (5/9) | 0.23 |
| 3^rd^ Cephalosporin resistant *** *K.* *pneumoniae* | 62.5%  (40/64) | 0%  (0/1) | 0.38 | 86.5%  (322/372) | 100%  (9/9) | 0.61 |
| Carbapenem resistant *** *E*. *coli* | 10,4%  (11/106) | 33.3%  (2/6) | 0.14 | 15%  (20/134) | 33.3%  (1/3) | 0.4 |
| 3^rd^ Cephalosporin resistant *** *E*. *coli* | 67%  (71/106) | 83.3%  (5/6) | 0.66 | 81.3%  (109/134) | 66.6%  (2/3) | 0.45 |
| Vancomycin resistant  *E*. *faecium* | 0% (0/1) | -  (0/0) | - | 9%  (1/11) | -  (0/0) | - |
| Methicillin resistant  *S*. *aureus* | 5.8%  (5/86) | 0%  (0/1) | >0.99 | 7.7%  (10/130) | 0%  (0/2) | >0.99 |
| Fluoroquinolone resistant  *Salmonella* spp | 11.1%  (3/27) | 0%  (0/1) | >0.99 | 16.6%  (3/18) | -  (0/0) | - |
| Fluoroquinolone resistant  *Shigella* spp | 100% (1/1) | -  (0/0) | - | -  (0/0) | -  (0/0) | - |
| Penicillin resistant  S. *pneumoniae* | 50%  (1/2) | 0%  (0/1) | >0.99 | -  (0/0) | -  (0/0) | - |
| Overall**** | 35%  (138/404) | 33.3%  (5/15) | 0.95 | 61.8%  (592/957) | 62%  (13/21) | 0.99 |

CO = community-origin. HO = hospital-origin. CO and HO are defined as proposed by WHO GLASS.(WHO, 2020) * Only the first pathogenic isolate per patient during the study period was included. ****** All COVID-19 cases were in 2020. *** All carbapenem-resistant *E. coli* and *K. pneumoniae* were also resistant to 3^rd^ cephalosporin. **** Among patients with blood culture positive for *Acinetobacter* spp., *P. aeruginosa*, *K.* *pneumoniae, E*. *coli, E*. *faecium, S*. *aureus, Salmonella* spp, *Shigella* spp or *S.* *pneumoniae*
