# Supplementary material for: Blood culture utilization and epidemiology of antimicrobial-resistant bloodstream infections before and during the COVID-19 pandemic in the Indonesian national referral hospital
Source: Antimicrob Resist Infect Control. 2022 May 19;11:73. doi: 10.1186/s13756-022-01114-x (PMC9117993; doi:10.1186/s13756-022-01114-x)
Supplement: Supplementary file 2 — Additional file2: Antimicrobial Resistance (AMR) Surveillance Report. Antimicrobial Resistance (AMR) Surveillance Report of the study site generated by AMASS [file 13756_2022_1114_MOESM2_ESM.pdf]

Antimicrobial Resistance (AMR) Surveillance Report

Hospital name: RSUPN Dr. Cipto Mangunkusumo
Country name: Indonesia

Data from:
01 Jan 2019 to 31 Dec 2020

Contact person: Robert Sinto
Contact address: Department of Internal Medicine, RSUPN Dr. Cipto Mangunkusumo
Contact email: robert.sinto01@ui.ac.id

Generated on: 26 Jan 2022

Generated by

AutoMated tool for Antimicrobial resistance Surveillance System (AMASS)
Version 1.1 (released on January 12, 2021)

The AMASS application is available under the Creative Commons Attribution 4.0 International Public License (CC BY 4.0). The application can be downloaded at: http://www.amass.website

The AMASS application used microbiology_data and hospital_admission_data files that are stored in the same folder as the application (AMASS.bat) to generate this report.

The goal of the AMASS application is to enable hospitals with microbiology data available in electronic formats to analyze their own data and generate AMR surveillance reports promptly. If hospital admission date data are available, the reports will additionally be stratified by infection origin (community−origin or hospital−origin). If mortality data (such as patient discharge outcome data) are available, a report on mortality involving AMR infection will be added.

This automatically generated report has limitations, and requires users to understand those limitations and use the summary data in the report with careful interpretation.

A valid report could have local implications and much wider benefits if shared with national and international organizations.

This automatically generated report is under the jurisdiction of the hospital to copy, redistribute, and share with any individual or organization.

This automatically generated report contains no patient identifier, similar to standard reports on cumulative antimicrobial susceptibility.

For any query on AMASS, please contact:
Cherry Lim (cherry@tropmedres.ac) and
Direk Limmathurotsakul (direk@tropmedres.ac)

Suggested title for citation:
Antimicrobial resistance surveillance report, RSUPN Dr. Cipto Mangunkusumo, Indonesia, NA to NA.

Content

Introduction 01

Section [1]: Data overview 03

Section [2]: Isolate−based surveillance report 05

Section [3]: Isolate−based surveillance report with stratification by infection origin 12

Section [4]: Sample−based surveillance report 24

Section [5]: Sample−based surveillance report with stratification by infection origin 27

Section [6]: Mortality involving AMR and antimicrobial−susuceptible infections 32

Methods 38

Acknowledgements 43

Introduction

Antimicrobial resistance (AMR) is a global health crisis [1]. The report by Lord Jim O'Neill estimated that 700,000 global deaths could be attributable to AMR in 2015, and projected that the annual death toll could reach 10 million by 2050 [1]. However, data of AMR surveillance from low and middle−income countries (LMICs) are scarce [1,2], and data of mortality associated with AMR infections are rarely available. A recent study estimated that 19,000 deaths are attributable to AMR infections in Thailand annually, using routinely available microbiological and hospital databases [3]. The study also proposed that hospitals in LMICs should utilize routinely available microbiological and hospital admission databases to generate reports on AMR surveillance systematically [3].

Reports on AMR surveillance can have a wide range of benefits [2]; including

- characterization of the frequency of resistance and organisms in different facilities and regions;
- prospective and retrospective information on emerging public health threats;
- evaluation and optimization of local and national standard treatment guidelines;
- evaluation of the impact of interventions beyond antimicrobial guidelines that aim to reduce AMR; and
- data sharing with national and international organizations to support decisions on resource allocation for interventions against AMR and to inform the implementation of action plans at national and global levels.

When reporting AMR surveillance results, it is generally recommended that (a) duplicate results of bacterial isolates are removed, and (b) reports are stratified by infection origin (community−origin or hospital−origin), if possible [2]. Many hospitals in LMICs lack time and resources needed to analyze the data (particularly to deduplicate data and to generate tables and figures), write the reports, and to release the data or reports [4].

AutoMated tool for Antimicrobial resistance Surveillance System (AMASS) was developed as an offline, open−access and easy−to−use application that allows a hospital to perform data analysis independently and generate isolate−based and sample−based surveillance reports stratified by infection origin from routinely collected electronic databases. The application was built in R, which is a free software environment. The application has been placed within a user−friendly interface that only requires the user to double−click on the application icon. The AMASS application can be downloaded at:
http://www.amass.website

Please note that the AMASS application and the automatically−generated report have limitations, and require readers to understand those limitations and review the reports and summary data carefully. We encourage the user of the AMASS application to perform manual validation (such as printing and listing isolates of the species to cross check with the reports), as recommended by Clinical and Laboratory Standards Insitute (CLSI) [5] and European Antimicrobial Resistance Surveillance Network (EUCAST) [6,7]. Moreover, it is important to note that the AMASS is an add−on automatized report generating tool and does not replace WHONET, Laboratory Information System (LIS), quality assurance programme, or antimicrobial surveillance systems (including the WHO GLASS).

References:

[1] O'Neill J. (2014) Antimicrobial resistance: tackling a crisis for the health and wealth of nations. Review on antimicrobial resistance. http://amr−review.org. (accessed on 3 Dec 2018).
[2] World Health Organization (2018) Global Antimicrobial Resistance Surveillance System (GLASS) Report. Early implantation 2016−2017. http://apps.who.int/iris/bitstream/handle/10665/259744/9789241513449−eng.pdf. (accessed on 3 Dec 2018)
[3] Lim C., et al. (2016) Epidemiology and burden of multidrug−resistant bacterial infection in a developing country. Elife 5: e18082.
[4] Ashley EA, Shetty N, Patel J, et al. Harnessing alternative sources of antimicrobial resistance data to support surveillance in low−resource settings. J Antimicrob Chemother. 2019; 74(3):541−546.
[5] Clinical and Laboratory Standards Institute (CLSI). Analysis and Presentation of Cumulative Antimicrobial Susceptibility Test Data, 4th Edition. 2014. (accessed on 21 Jan 2020)
[6] European Antimicrobial Resistance Surveillance Network (EARS−Net). Antimicrobial resistance (AMR) reporting protocol 2018. (accessed on 21 Jan 2020)
[7] European Committee on Antimicrobial Susceptibility Testing (EUCAST). www.eucast.org (accessed on 21 Jan 2020)

Section [1]: Data overview

Introduction

An overview of the data detected by the AMASS application is generated by default. The summary is based on the raw data files saved within the same folder as the application file (AMASS.bat).

Please review and validate this section carefully before proceeds to the next section.

Results

The microbiology_data file (stored in the same folder as the application file) had:

17901 specimen data records with collection dates ranging from ***01 Jan 2019*** to ***31 Dec 2020***

The hospital_admission_data file (stored in the same folder as the application file) had:

91960 admission data records with hospital admission dates ranging from ***NA*** to ***NA***

Notes:

[1] If the periods of the data in microbiology_data and hospital_admission_data files are not similar, the automatically−generated report should be interpreted with caution. The AMASS generates the reports based on the available data.

Reporting period by months:

Data was stratified by month to assist detection of missing data, and verification of whether the month distribution of data records in microbiology_data file and hospital_admission_data file reflected the microbiology culture frequency and admission rate of the hospital, respectively. For example if the number of specimens in the microbiology_data file reported below is lower than what is expected, please check the raw data file and data dictionary files.

Month	Number of specimen data records in microbiology_data file	Number of admission data records in hospital_admission_data file
January	*1235*	*8037*
February	*1465*	*7431*
March	*1414*	*7730*
April	*1339*	*6025*
May	*1400*	*6551*
June	*1443*	*6761*
July	*1561*	*8098*
August	*1571*	*8518*
September	*1448*	*8007*
October	*1612*	*7947*
November	*1670*	*8381*
December	*1743*	*8474*
Total:	*17901*	*91960*

Note:

[1] Additional general demographic data will be made available in the next version of the AMASS application.

Section [2]: Isolate−based surveillance report

Introduction
An isolate−based surveillance report is generated by default, even if the hospital_admission_data file is unavailable. This is to enable hospitals with only microbiology data available to utilize the de−duplication and report generation functions of AMASS.
This report is without stratification by origin of infection.

The report generated by the AMASS application version 1.1 includes only blood samples. The next version of AMASS will include other specimen types, including cerebrospinal fluid (CSF), urine, stool, and other specimens.

Organisms under this survey:

− *Staphylococcus aureus*
− *Enterococcus* spp.
− *Streptococcus pneumoniae*
− *Salmonella* spp.
− *Escherichia coli*
− *Klebsiella pneumoniae*
− *Pseudomonas aeruginosa*
− *Acinetobacter* spp.

Results
The microbiology_data file had:

Sample collection dates ranged from ***01 Jan 2019*** to ***31 Dec 2020***
Number of records of blood specimens collected within the above date range:
17901 blood specimens records

*Number of records of blood specimens with *negative culture (no growth):*
14205 blood specimens records
Number of records of blood specimens with culture positive for a microorganism:
3696 blood specimens records

Number of records of blood specimens with culture positive for organism under this survey:
2030 blood specimens records

The AMASS application de−duplicated the data by including only the first isolate per patient per specimen type per evaluation period as described in the method. The number of patients with positive samples is as follows:

**Organism**	**Number of records of blood specimens culture positive for the organism**	****Number of patients with blood culture positive for the organism (de−duplicated)**
Staphylococcus aureus	***293***	***212***
***Enterococcus* spp.**	***90***	***74***
Streptococcus pneumoniae	***3***	***3***
***Salmonella* spp.**	***49***	***45***
Escherichia coli	***288***	***248***
Klebsiella pneumoniae	***738***	***441***
Pseudomonas aeruginosa	***208***	***160***
***Acinetobacter* spp.**	***361***	***249***
**Total:**	***2030***	***1432***

*The negative culture included data values specified as 'no growth' in the dictionary_for_ microbiology_data file (details on data dictionary files are in the method section) to represent specimens with negative culture for any microorganism.
**Only the first isolate for each patient per specimen type, per pathogen, and per evaluation period was included in the analysis.

The following figures and tables show the proportion of patients with blood culture positive for antimicrobial non−susceptible isolates.

Section [2]: Isolate−based surveillance report

Blood: *Staphylococcus aureus* ***(No. of patients = 212)***

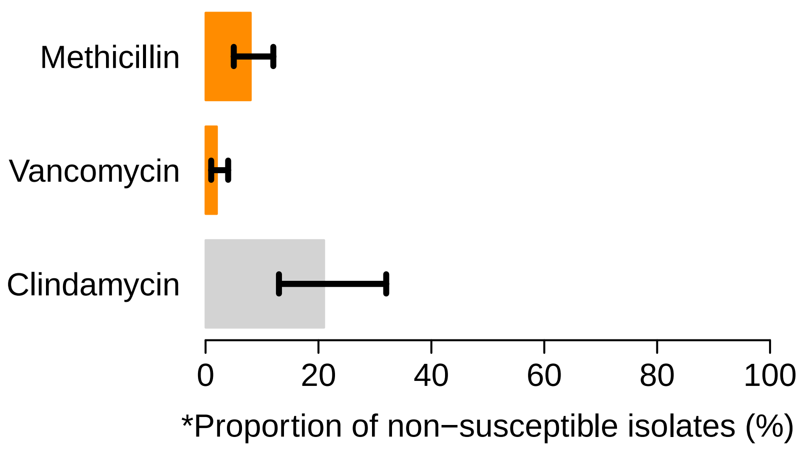

Antibiotic agent	% NS (n)	95% CI
Methicillin	8% (16/209)	5%−12%
Vancomycin	2% (3/193)	1%−4%
Clindamycin	21% (15/70)	13%−32%

Blood: *Enterococcus* spp. ***(No. of patients = 74)***

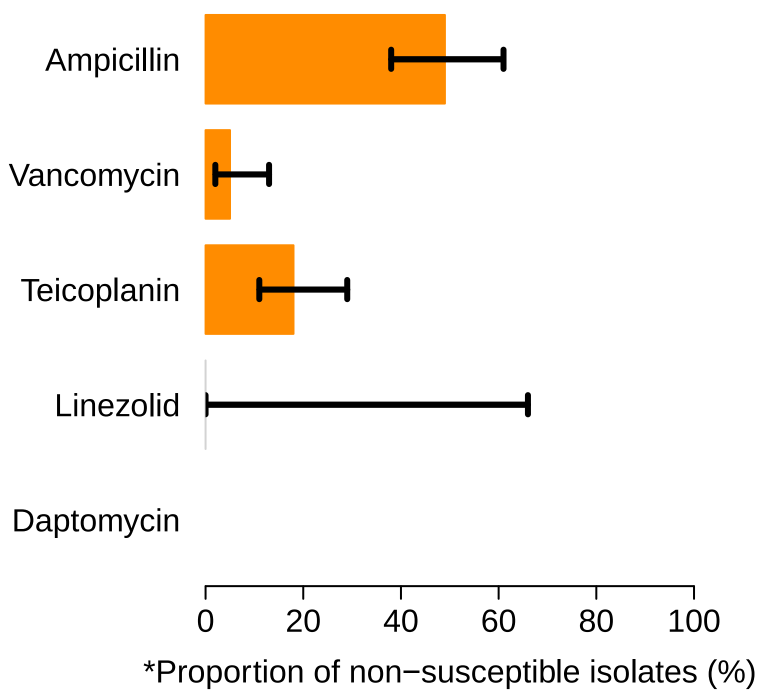

Antibiotic agent	% NS (n)	95% CI
Ampicillin	49% (35/71)	38%−61%
Vancomycin	5% (3/61)	2%−13%
Teicoplanin	18% (12/67)	11%−29%
Linezolid	0% (0/2)	0%−66%
Daptomycin	NA	−

*Proportion of non−susceptible isolates (% NS) represents the number of patients with blood culture positive for non−susceptible isolates (numerator) over the total number of patients with blood culture positive for the organism and the organism was tested for susceptibility against the antibiotic (denominator). The AMASS application de−duplicated the data by including only the first isolate per patient per specimen type per evaluation period. Grey bars indicate that testing with the antibiotic occurred for less than 70% of the total number of patients with blood culture positive for the organism.
CI = confidence interval; NA = Not available/reported/tested; Methicillin: methicillin, oxacillin, or cefoxitin

Section [2]: Isolate−based surveillance report

Blood: *Streptococcus pneumoniae*

(No. of patients = 3)

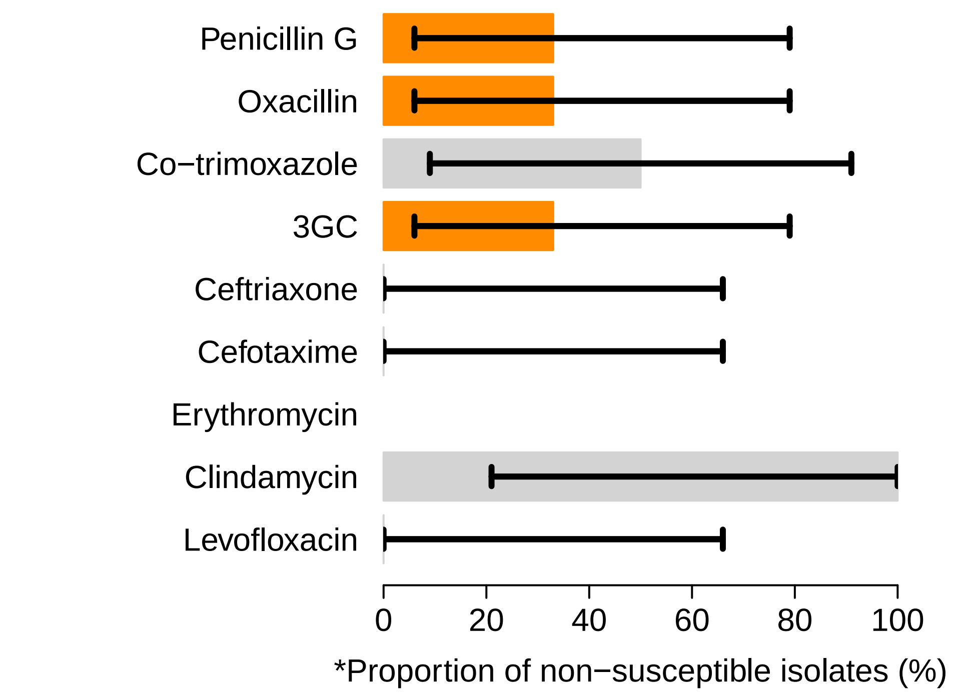

Antibiotic agent	% NS (n)	95% CI
Penicillin G	33% (1/3)	6%−79%
Oxacillin	33% (1/3)	6%−79%
Co−trimoxazole	50% (1/2)	9%−91%
3GC	33% (1/3)	6%−79%
Ceftriaxone	0% (0/2)	0%−66%
Cefotaxime	0% (0/2)	0%−66%
Erythromycin	NA	−
Clindamycin	100% (1/1)	21%−100%
Levofloxacin	0% (0/2)	0%−66%

Blood: *Salmonella* spp.

(No. of patients = 45)

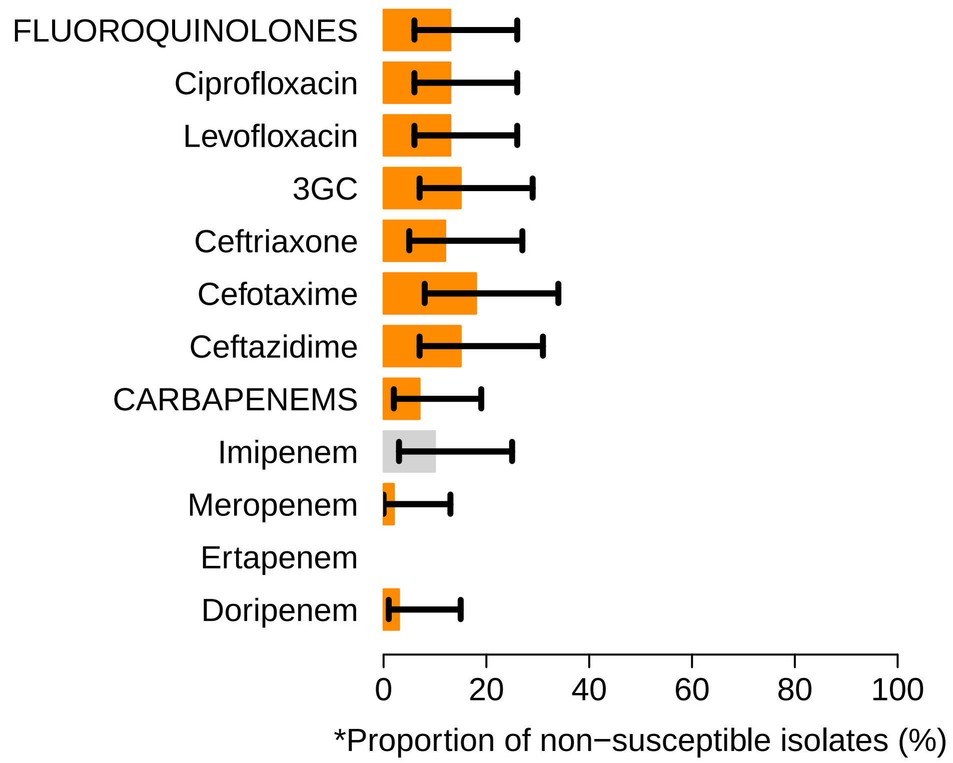

Antibiotic agent	% NS (n)	95% CI
FLUOROQUINOLONES	13% (6/45)	6%−26%
Ciprofloxacin	13% (6/45)	6%−26%
Levofloxacin	13% (6/45)	6%−26%
3GC	15% (6/40)	7%−29%
Ceftriaxone	12% (4/34)	5%−27%
Cefotaxime	18% (6/34)	8%−34%
Ceftazidime	15% (5/33)	7%−31%
CARBAPENEMS	7% (3/43)	2%−19%
Imipenem	10% (3/31)	3%−25%
Meropenem	2% (1/40)	0%−13%
Ertapenem	NA	−
Doripenem	3% (1/35)	1%−15%

*Proportion of non−susceptible isolates (% NS) represents the number of patients with blood culture positive for non−susceptible isolates (numerator) over the total number of patients with blood culture positive for the organism and the organism was tested for susceptibility against the antibiotic (denominator). The AMASS application de−duplicated the data by including only the first isolate per patient per specimen type per evaluation period. Grey bars indicate that testing with the antibiotic occurred for less than 70% of the total number of blood culture positive for the organism.
CI = confidence interval; NA = Not available/reported/tested; 3GC = 3rd−generation cephalosporin;
FLUOROQUINOLONES: ciprofloxacin or levofloxacin; CARBAPENEMS: imipenem, meropenem, ertapenem or doripenem

Section [2]: Isolate−based surveillance report

Blood: ***Escherichia coli*** ***(No. of patients = 248)***

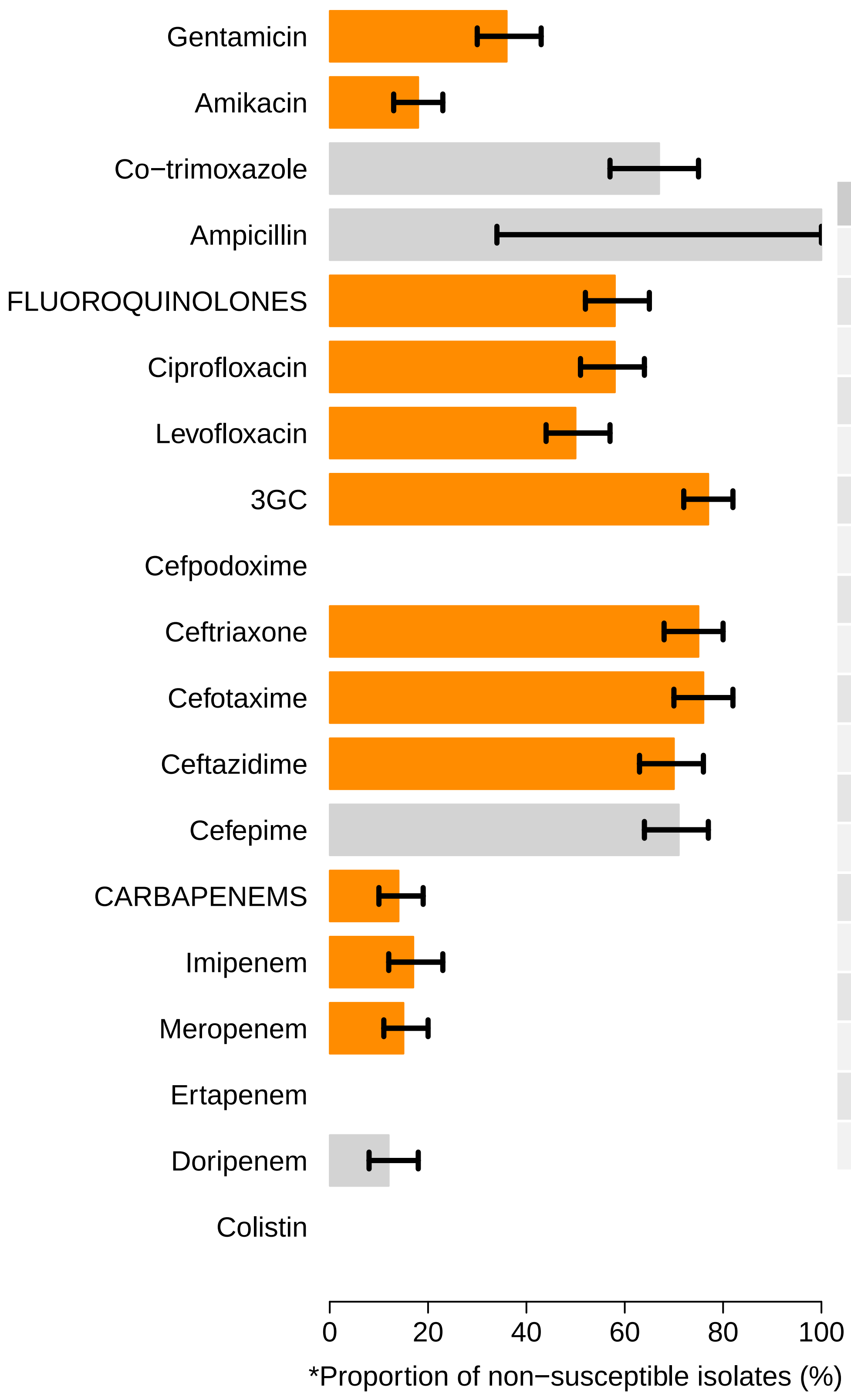

Antibiotic agent	% NS (n)	95% CI
Gentamicin	36% (80/221)	30%−43%
Amikacin	18% (41/230)	13%−23%
Co−trimoxazole	67% (68/102)	57%−75%
Ampicillin	100% (2/2)	34%−100%
FLUOROQUINOLONES	58% (130/223)	52%−65%
Ciprofloxacin	58% (126/219)	51%−64%
Levofloxacin	50% (103/204)	44%−57%
3GC	77% (187/242)	72%−82%
Cefpodoxime	NA	−
Ceftriaxone	75% (153/205)	68%−80%
Cefotaxime	76% (155/203)	70%−82%
Ceftazidime	70% (126/181)	63%−76%
Cefepime	71% (115/162)	64%−77%
CARBAPENEMS	14% (34/239)	10%−19%
Imipenem	17% (32/186)	12%−23%
Meropenem	15% (31/208)	11%−20%
Ertapenem	NA	−
Doripenem	12% (19/162)	8%−18%
Colistin	NA	−

*Proportion of non−susceptible isolates (% NS) represents the number of patients with blood culture positive for non−susceptible isolates (numerator) over the total number of patients with blood culture positive for the organism and the organism was tested for susceptibility against the antibiotic (denominator). The AMASS application de−duplicated the data by including only the first isolate per patient per specimen type per evaluation period. Grey bars indicate that testing with the antibiotic occurred for less than 70% of the total number of blood culture positive for the organism.
CI = confidence interval; NA = Not available/reported/tested; 3GC = 3rd−generation cephalosporin;
FLUOROQUINOLONES: ciprofloxacin or levofloxacin; CARBAPENEMS: imipenem, meropenem, ertapenem or doripenem

Section [2]: Isolate−based surveillance report

Blood: ***Klebsiella pneumoniae*** ***(No. of patients = 441)***

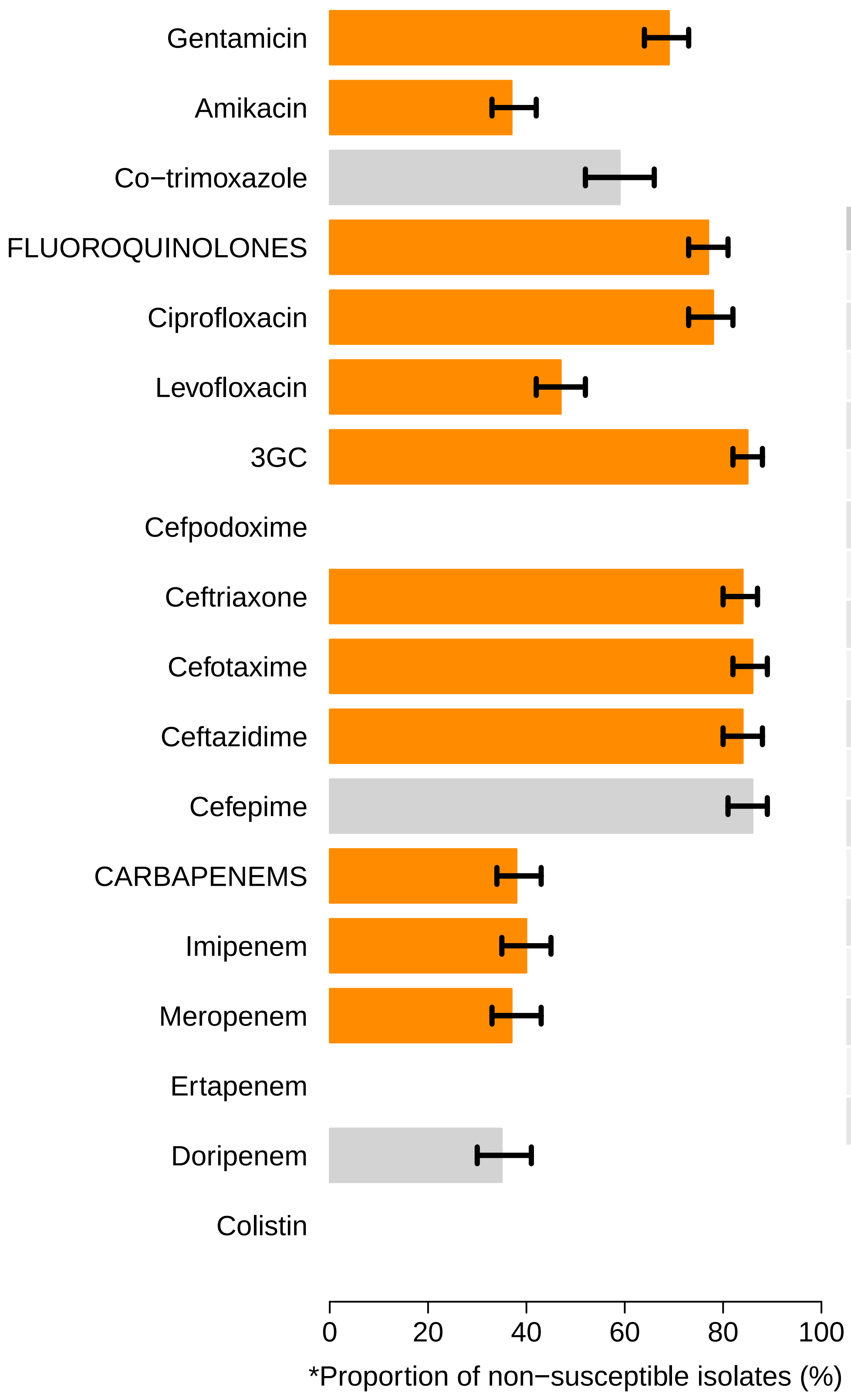

Antibiotic agent	% NS (n)	95% CI
Gentamicin	69% (268/388)	64%−73%
Amikacin	37% (150/402)	33%−42%
Co−trimoxazole	59% (115/194)	52%−66%
FLUOROQUINOLONES	77% (291/376)	73%−81%
Ciprofloxacin	78% (279/358)	73%−82%
Levofloxacin	47% (165/351)	42%−52%
3GC	85% (371/434)	82%−88%
Cefpodoxime	NA	−
Ceftriaxone	84% (310/369)	80%−87%
Cefotaxime	86% (287/334)	82%−89%
Ceftazidime	84% (277/330)	80%−88%
Cefepime	86% (240/280)	81%−89%
CARBAPENEMS	38% (160/419)	34%−43%
Imipenem	40% (126/315)	35%−45%
Meropenem	37% (134/358)	33%−43%
Ertapenem	NA	−
Doripenem	35% (99/284)	30%−41%
Colistin	NA	−

*Proportion of non−susceptible isolates (% NS) represents the number of patients with blood culture positive for non−susceptible isolates (numerator) over the total number of patients with blood culture positive for the organism and the organism was tested for susceptibility against the antibiotic (denominator). The AMASS application de−duplicated the data by including only the first isolate per patient per specimen type per evaluation period. Grey bars indicate that testing with the antibiotic occurred for less than 70% of the total number of blood culture positive for the organism.
CI = confidence interval; NA = Not available/reported/tested; 3GC = 3rd−generation cephalosporin; FLUOROQUINOLONES: ciprofloxacin or levofloxacin; CARBAPENEMS: imipenem, meropenem, ertapenem or doripenem

Section [2]: Isolate−based surveillance report

Blood: *Pseudomonas aeruginosa* (No. of patients = 160)

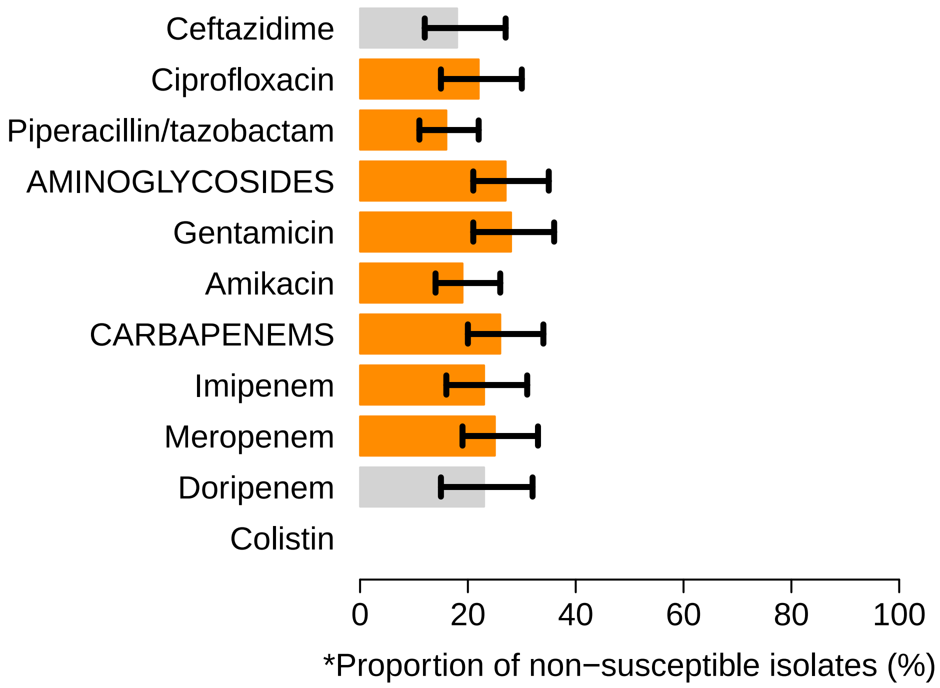

Antibiotic agent	% NS (n)	95% CI
Ceftazidime	18% (19/105)	12%−27%
Ciprofloxacin	22% (27/125)	15%−30%
Piperacillin/tazobactam	16% (23/147)	11%−22%
AMINOGLYCOSIDES	27% (42/153)	21%−35%
Gentamicin	28% (38/136)	21%−36%
Amikacin	19% (28/145)	14%−26%
CARBAPENEMS	26% (40/153)	20%−34%
Imipenem	23% (28/122)	16%−31%
Meropenem	25% (33/131)	19%−33%
Doripenem	23% (22/97)	15%−32%
Colistin	NA	−

Blood: *Acinetobacter* spp. (No. of patients = 249)

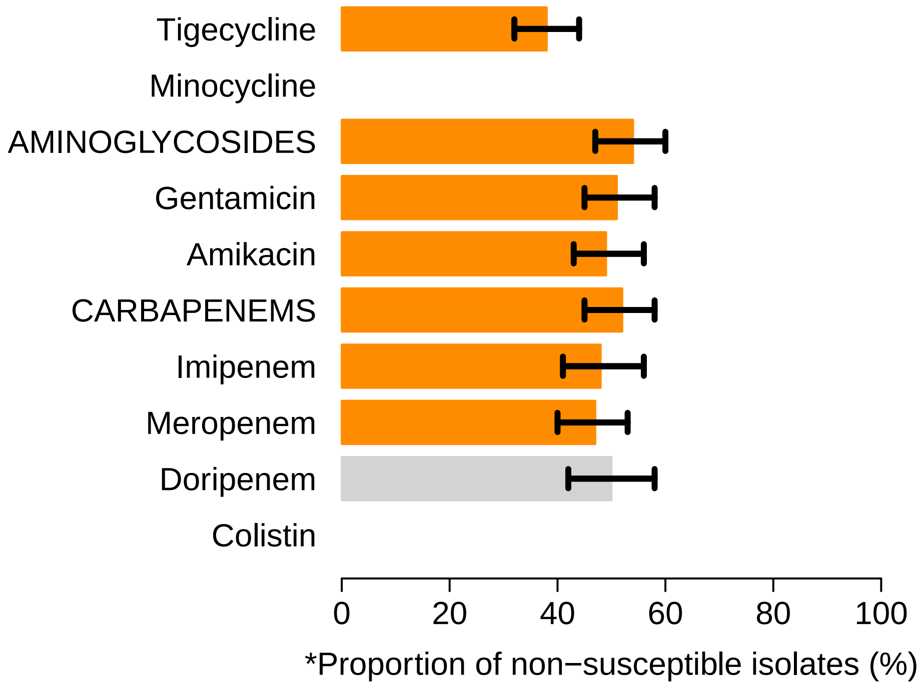

Antibiotic agent	% NS (n)	95% CI
Tigecycline	38% (83/220)	32%−44%
Minocycline	NA	−
AMINOGLYCOSIDES	54% (125/232)	47%−60%
Gentamicin	51% (107/208)	45%−58%
Amikacin	49% (108/219)	43%−56%
CARBAPENEMS	52% (120/233)	45%−58%
Imipenem	48% (85/176)	41%−56%
Meropenem	47% (94/202)	40%−53%
Doripenem	50% (77/154)	42%−58%
Colistin	NA	−

*Proportion of non−susceptible isolates (% NS) represents the number of patients with blood culture positive for non−susceptible isolates (numerator) over the total number of patients with blood culture positive for the organism and the organism was tested for susceptibility against the antibiotic (denominator). The AMASS application de−duplicated the data by including only the first isolate per patient per specimen type per evaluation period. Grey bars indicate that testing with the antibiotic occurred for less than 70% of the total number of blood culture positive for the organism.
CI = confidence interval; NA = Not available/reported/tested; AMINOGLYCOSIDES: either gentamicin or amikacin; CARBAPENEMS: imipenem, meropenem, ertapenem or doripenem

Section [3]: Isolate−based surveillance report with stratification by infection origin

Introduction

An isolate−based surveillance report with stratification by origin of infection is generated only if admission date data are available in the raw data file(s) with the appropriate specification in the data dictionaries.

Stratification by origin of infection is used as a proxy to define where the bloodstream infection (BSI) was contracted (hospital versus community).

The definitions of infection origin proposed by the WHO GLASS are used. In brief, community−origin BSI is defined as patients in the hospital for less than or equal to two calendar days when the first specimen culture postive for the pathogen was taken. Hospital−origin BSI is defined as patients admitted for more than two calendar days when the first specimen culture positive for the pathogen was taken.

Results:

The data included in the analysis to generate the report had:

Sample collection dates ranged from ***01 Jan 2019*** to ***31 Dec 2020***

**Number of patients with blood culture positive for pathogen under the survey:*

1432 patients

***Number of patients with community−origin BSI:*

418 patients

***Number of patients with hospital−origin BSI:*

1014 patients

****Number of patients with unknown infection of origin status:*

0 patients

Organism	Number of patients with blood culture positive for the organism	Community −origin**	Hospital −origin**	Unknown origin***
Staphylococcus aureus	***212***	***82***	***130***	***0***
***Enterococcus* spp.**	***74***	***17***	***57***	***0***
Streptococcus pneumoniae	***3***	***3***	***0***	***0***
***Salmonella* spp.**	***45***	***27***	***18***	***0***
Escherichia coli	***248***	***111***	***137***	***0***
Klebsiella pneumoniae	***441***	***65***	***376***	***0***
Pseudomonas aeruginosa	***160***	***60***	***100***	***0***
***Acinetobacter* spp.**	***249***	***53***	***196***	***0***
**Total:**	**1432**	***418***	***1014***	***0***

Note:
NA= Not applicable (hospital admission date or infection origin data are not available)

*Only the first isolate for each patient per specimen type per pathogen under the reporting period is included in the analysis. Please refer to Section [2] for details on how this number was calculated from the raw microbiology_data file.
**The definitions of infection origin proposed by the WHO GLASS is used. In brief, community−origin BSI was defined as patients in the hospital for less than or equal to two calendar days when the first blood culture positive for the pathogen was taken. Hospital−origin BSI was defined as patients admitted for more than two calendar days when the first specimen culture positive for the pathogen was taken.
Please refer to the 'Methods' section for more details on the definitions used.
***Unknown origin could be because admission date data are not available or the patient was not hospitalised.

The following figures and tables below show the proportion of patients with blood culture positive for antimicrobial non−susceptible isolates stratified by infection of origin.

Section [3]: Isolate−based surveillance report with stratification by infection origin

Blood: ***Staphylococcus aureus*** **Community−origin** ***(No. of patients = 82)***

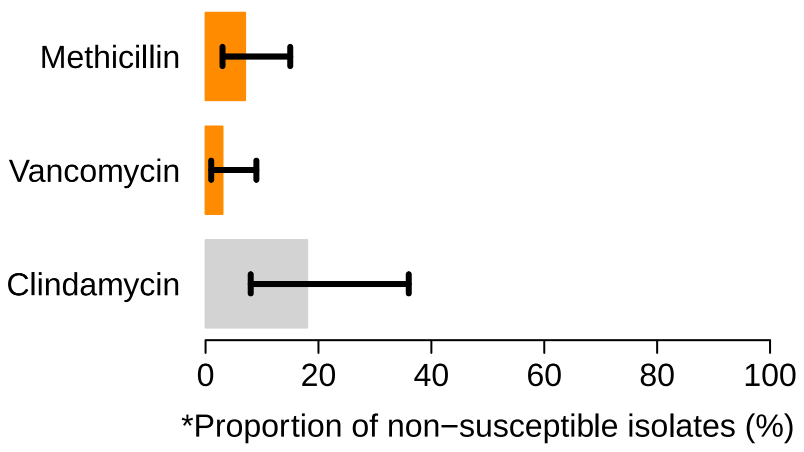

Antibiotic agent	% NS (n)	95% CI
Methicillin	7% (6/82)	3%−15%
Vancomycin	3% (2/74)	1%−9%
Clindamycin	18% (5/28)	8%−36%

Blood: ***Staphylococcus aureus*** **Hospital−origin** ***(No. of patients = 130)***

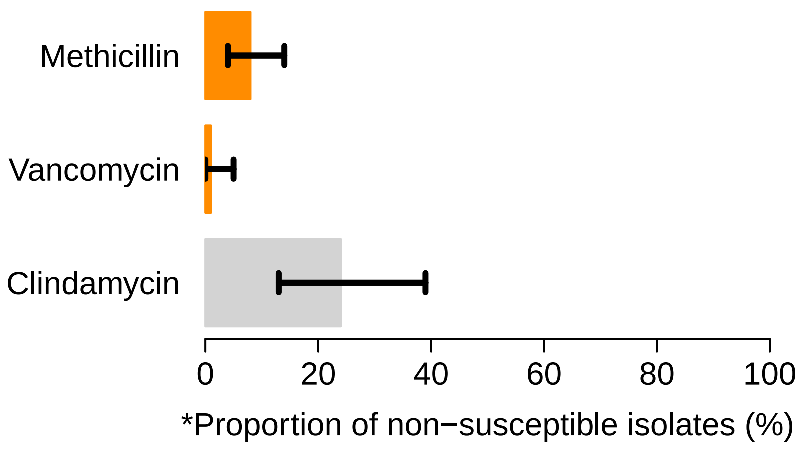

Antibiotic agent	% NS (n)	95% CI
Methicillin	8% (10/127)	4%−14%
Vancomycin	1% (1/119)	0%−5%
Clindamycin	24% (10/42)	13%−39%

*Proportion of non−susceptible isolates (% NS) represents the number of patients with blood culture positive for non−susceptible isolates (numerator) over the total number of patients with blood culture positive for the organism and the organism was tested for susceptibility against the antibiotic (denominator). The AMASS application de−duplicated the data by including only the first isolate per patient per specimen type per evaluation period. Grey bars indicate that testing with the antibiotic occurred for less than 70% of the total number of patients with blood culture positive for the organism.
CI = confidence interval; NA = Not available/reported/tested; Methicillin: methicillin, oxacillin, or cefoxitin

Section [3]: Isolate−based surveillance report with stratification by infection origin

Blood: *Enterococcus* spp. Community−origin (No. of patients = 17)

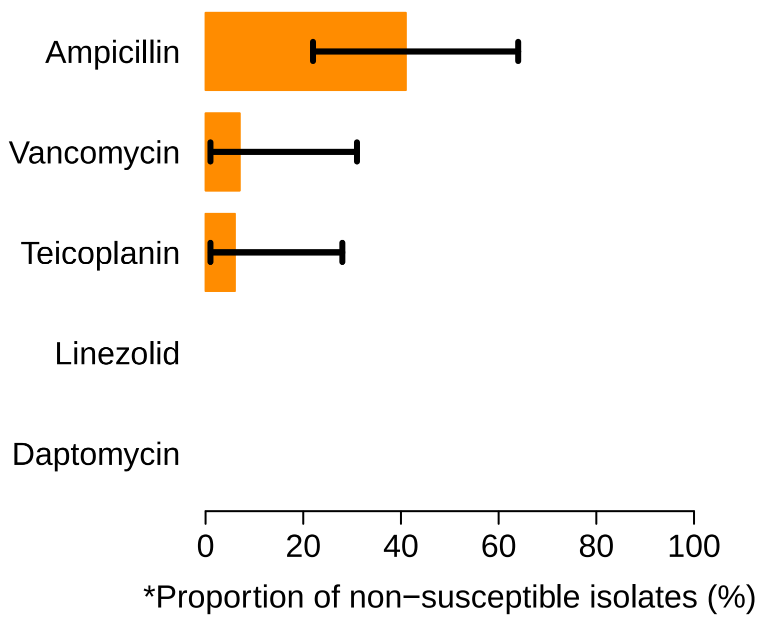

Antibiotic agent	% NS (n)	95% CI
Ampicillin	41% (7/17)	22%−64%
Vancomycin	7% (1/14)	1%−31%
Teicoplanin	6% (1/16)	1%−28%
Linezolid	NA	−
Daptomycin	NA	−

Blood: *Enterococcus* spp. Hospital−origin (No. of patients = 57)

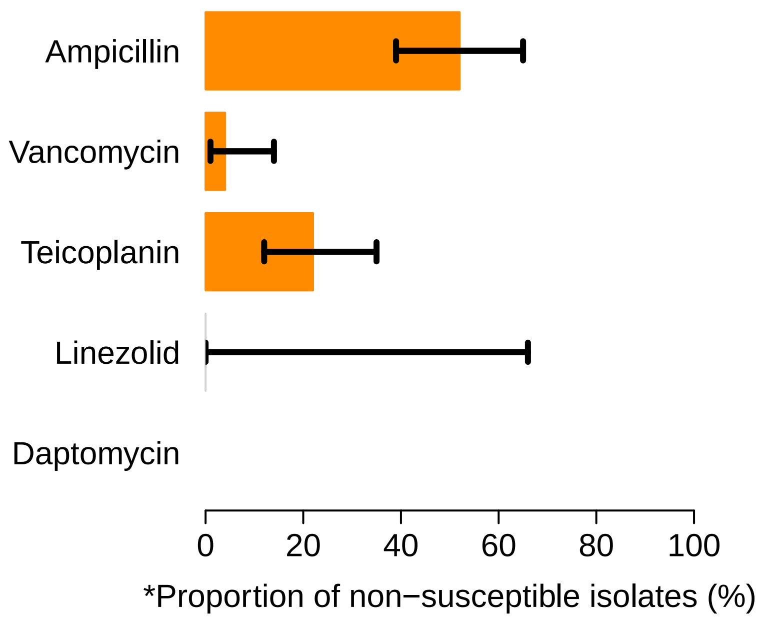

Antibiotic agent	% NS (n)	95% CI
Ampicillin	52% (28/54)	39%−65%
Vancomycin	4% (2/47)	1%−14%
Teicoplanin	22% (11/51)	12%−35%
Linezolid	0% (0/2)	0%−66%
Daptomycin	NA	−

*Proportion of non−susceptible isolates (% NS) represents the number of patients with blood culture positive for non−susceptible isolates (numerator) over the total number of patients with blood culture positive for the organism and the organism was tested for susceptibility against the antibiotic (denominator). The AMASS application de−duplicated the data by including only the first isolate per patient per specimen type per evaluation period. Grey bars indicate that testing with the antibiotic occurred for less than 70% of the total number of patients with blood culture positive for the organism.
CI = confidence interval; NA = Not available/reported/tested; Methicillin: methicillin, oxacillin, or cefoxitin

Section [3]: Isolate−based surveillance report with stratification by infection origin

Blood: *Streptococcus pneumoniae* Community−origin ***(No. of patients = 3)***

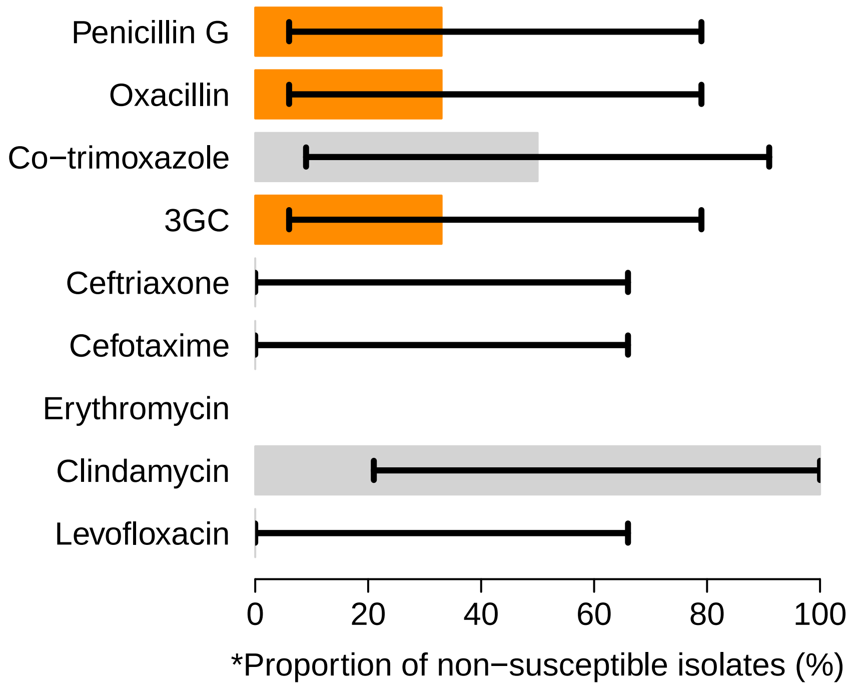

Antibiotic agent	% NS (n)	95% CI
Penicillin G	33% (1/3)	6%−79%
Oxacillin	33% (1/3)	6%−79%
Co−trimoxazole	50% (1/2)	9%−91%
3GC	33% (1/3)	6%−79%
Ceftriaxone	0% (0/2)	0%−66%
Cefotaxime	0% (0/2)	0%−66%
Erythromycin	NA	−
Clindamycin	100% (1/1)	21%−100%
Levofloxacin	0% (0/2)	0%−66%

Blood: *Streptococcus pneumoniae* Hospital−origin ***(No. of patients = 0)***

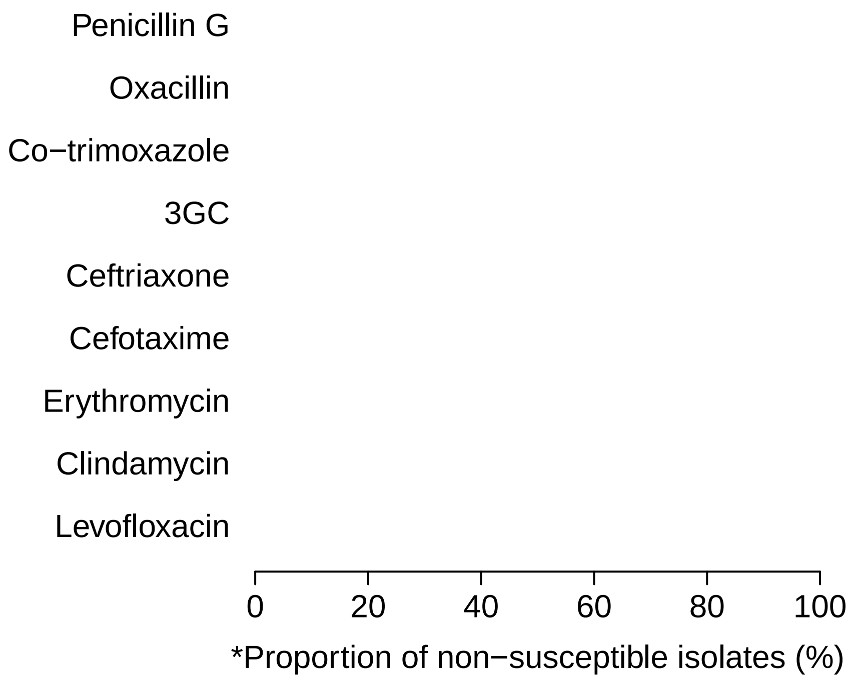

Antibiotic agent	% NS (n)	95% CI
Penicillin G	NA	−
Oxacillin	NA	−
Co−trimoxazole	NA	−
3GC	NA	−
Ceftriaxone	NA	−
Cefotaxime	NA	−
Erythromycin	NA	−
Clindamycin	NA	−
Levofloxacin	NA	−

*Proportion of non−susceptible isolates (% NS) represents the number of patients with blood culture positive for non−susceptible isolates (numerator) over the total number of patients with blood culture positive for the organism and the organism was tested for susceptibility against the antibiotic (denominator). The AMASS application de−duplicated the data by including only the first isolate per patient per specimen type per evaluation period. Grey bars indicate that testing with the antibiotic occurred for less than 70% of the total number of blood culture positive for the organism.
CI = confidence interval; NA = Not available/reported/tested; 3GC = 3rd−generation cephalosporin; FLUOROQUINOLONES: ciprofloxacin or levofloxacin; CARBAPENEMS: imipenem, meropenem, ertapenem or doripenem

Section [3]: Isolate−based surveillance report with stratification by infection origin

Blood: ***Salmonella* spp.** **Community−origin** ***(No. of patients = 27)***

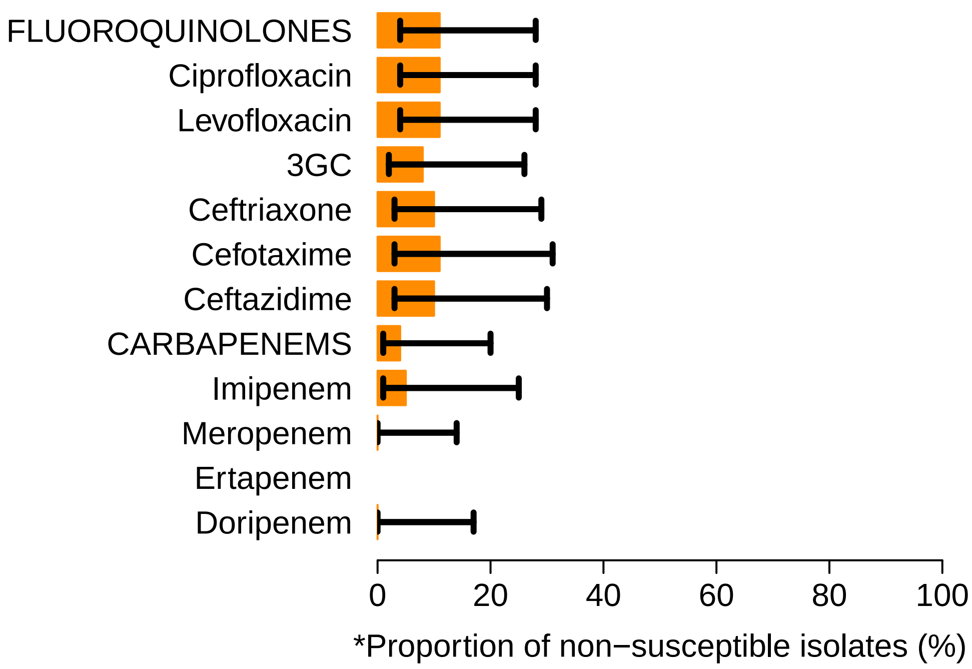

Antibiotic agent	% NS (n)	95% CI
FLUOROQUINOLONES	11% (3/27)	4%−28%
Ciprofloxacin	11% (3/27)	4%−28%
Levofloxacin	11% (3/27)	4%−28%
3GC	8% (2/24)	2%−26%
Ceftriaxone	10% (2/21)	3%−29%
Cefotaxime	11% (2/19)	3%−31%
Ceftazidime	10% (2/20)	3%−30%
CARBAPENEMS	4% (1/25)	1%−20%
Imipenem	5% (1/19)	1%−25%
Meropenem	0% (0/24)	0%−14%
Ertapenem	NA	−
Doripenem	0% (0/19)	0%−17%

Blood: ***Salmonella* spp.** **Hospital−origin** ***(No. of patients = 18)***

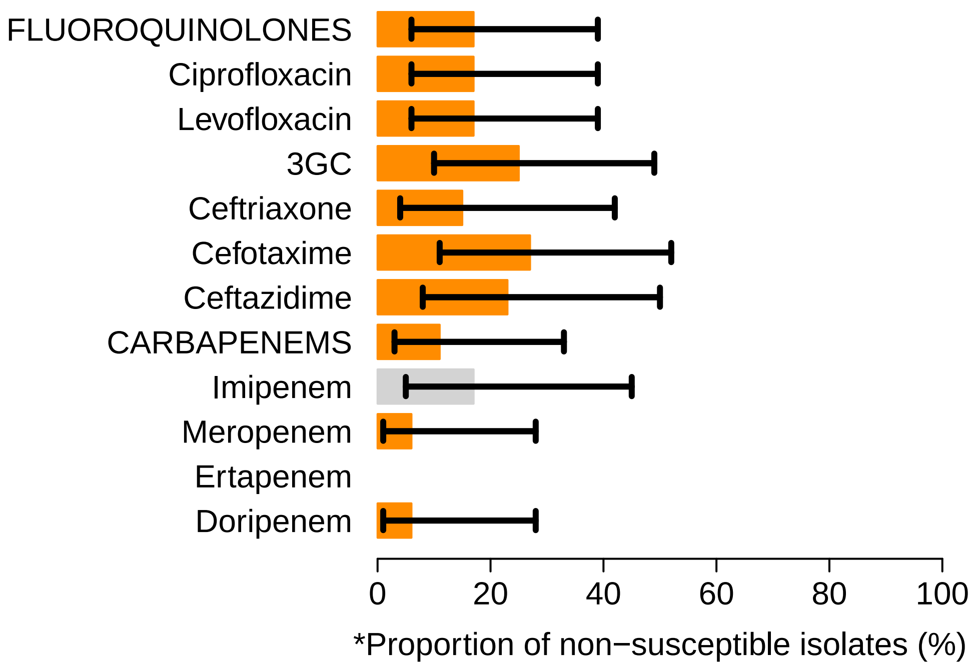

Antibiotic agent	% NS (n)	95% CI
FLUOROQUINOLONES	17% (3/18)	6%−39%
Ciprofloxacin	17% (3/18)	6%−39%
Levofloxacin	17% (3/18)	6%−39%
3GC	25% (4/16)	10%−49%
Ceftriaxone	15% (2/13)	4%−42%
Cefotaxime	27% (4/15)	11%−52%
Ceftazidime	23% (3/13)	8%−50%
CARBAPENEMS	11% (2/18)	3%−33%
Imipenem	17% (2/12)	5%−45%
Meropenem	6% (1/16)	1%−28%
Ertapenem	NA	−
Doripenem	6% (1/16)	1%−28%

*Proportion of non−susceptible isolates (% NS) represents the number of patients with blood culture positive for non−susceptible isolates (numerator) over the total number of patients with blood culture positive for the organism and the organism was tested for susceptibility against the antibiotic (denominator). The AMASS application de−duplicated the data by including only the first isolate per patient per specimen type per evaluation period. Grey bars indicate that testing with the antibiotic occurred for less than 70% of the total number of blood culture positive for the organism.
CI = confidence interval; NA = Not available/reported/tested; 3GC = 3rd−generation cephalosporin; FLUOROQUINOLONES: ciprofloxacin or levofloxacin; CARBAPENEMS: imipenem, meropenem, ertapenem or doripenem

Section [3]: Isolate−based surveillance report with stratification by infection origin

Blood: ***Escherichia coli***

Community−origin ***(No. of patients = 111)***

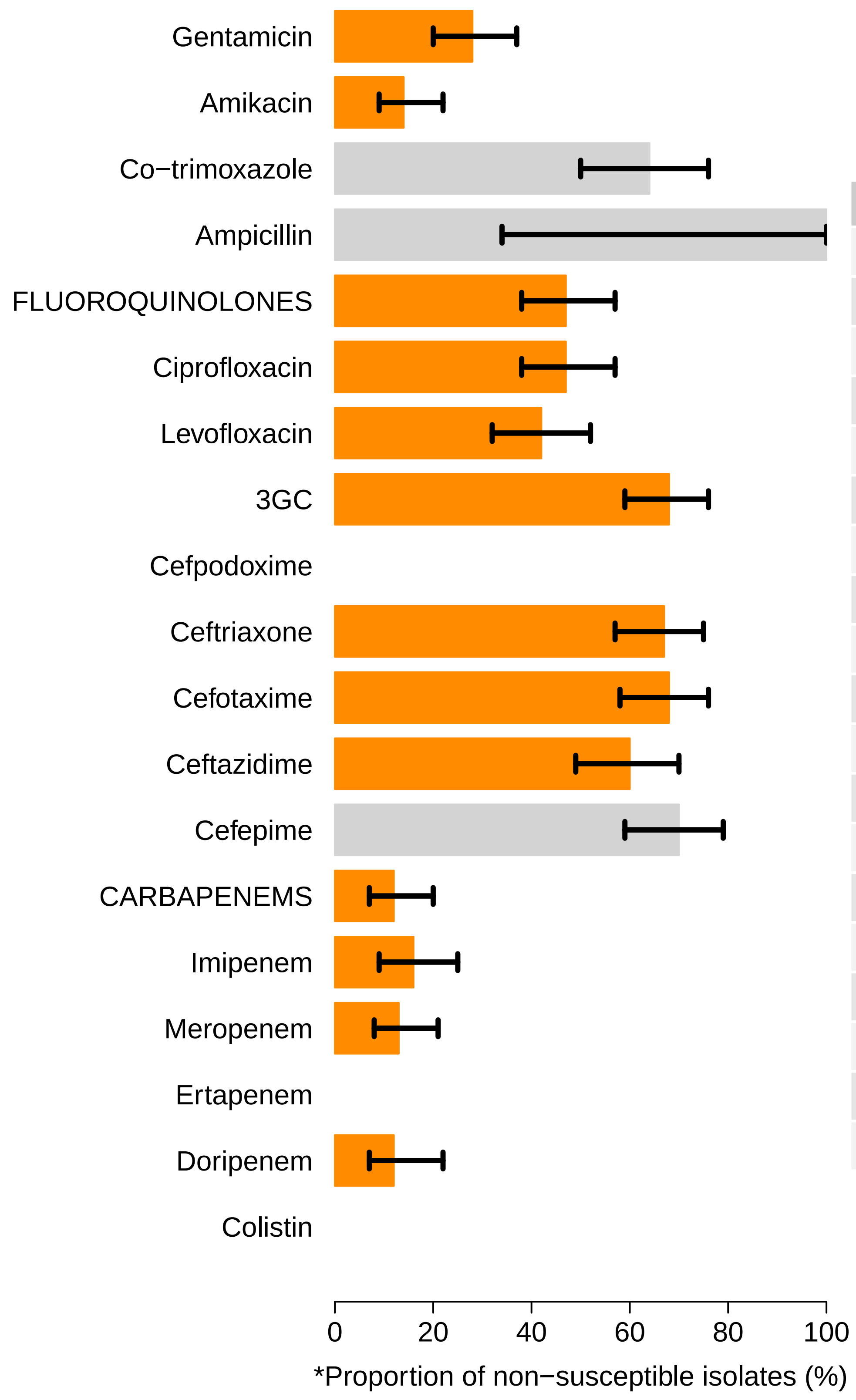

Antibiotic agent	% NS (n)	95% CI
Gentamicin	28% (28/101)	20%−37%
Amikacin	14% (15/105)	9%−22%
Co−trimoxazole	64% (30/47)	50%−76%
Ampicillin	100% (2/2)	34%−100%
FLUOROQUINOLONES	47% (49/104)	38%−57%
Ciprofloxacin	47% (48/102)	38%−57%
Levofloxacin	42% (40/96)	32%−52%
3GC	68% (76/111)	59%−76%
Cefpodoxime	NA	−
Ceftriaxone	67% (62/93)	57%−75%
Cefotaxime	68% (66/97)	58%−76%
Ceftazidime	60% (50/83)	49%−70%
Cefepime	70% (54/77)	59%−79%
CARBAPENEMS	12% (13/107)	7%−20%
Imipenem	16% (13/83)	9%−25%
Meropenem	13% (12/93)	8%−21%
Ertapenem	NA	−
Doripenem	12% (10/80)	7%−22%
Colistin	NA	−

*Proportion of non−susceptible isolates (% NS) represents the number of patients with blood culture positive for non−susceptible isolates (numerator) over the total number of patients with blood culture positive for the organism and the organism was tested for susceptibility against the antibiotic (denominator). The AMASS application de−duplicated the data by including only the first isolate per patient per specimen type per evaluation period. Grey bars indicate that testing with the antibiotic occurred for less than 70% of the total number of blood culture positive for the organism.
CI = confidence interval; NA = Not available/reported/tested; 3GC = 3rd−generation cephalosporin; FLUOROQUINOLONES: ciprofloxacin or levofloxacin; CARBAPENEMS: imipenem, meropenem, ertapenem or doripenem

Section [3]: Isolate−based surveillance report with stratification by infection origin

Blood: ***Escherichia coli*** **Hospital−origin** ***(No. of patients = 137)***

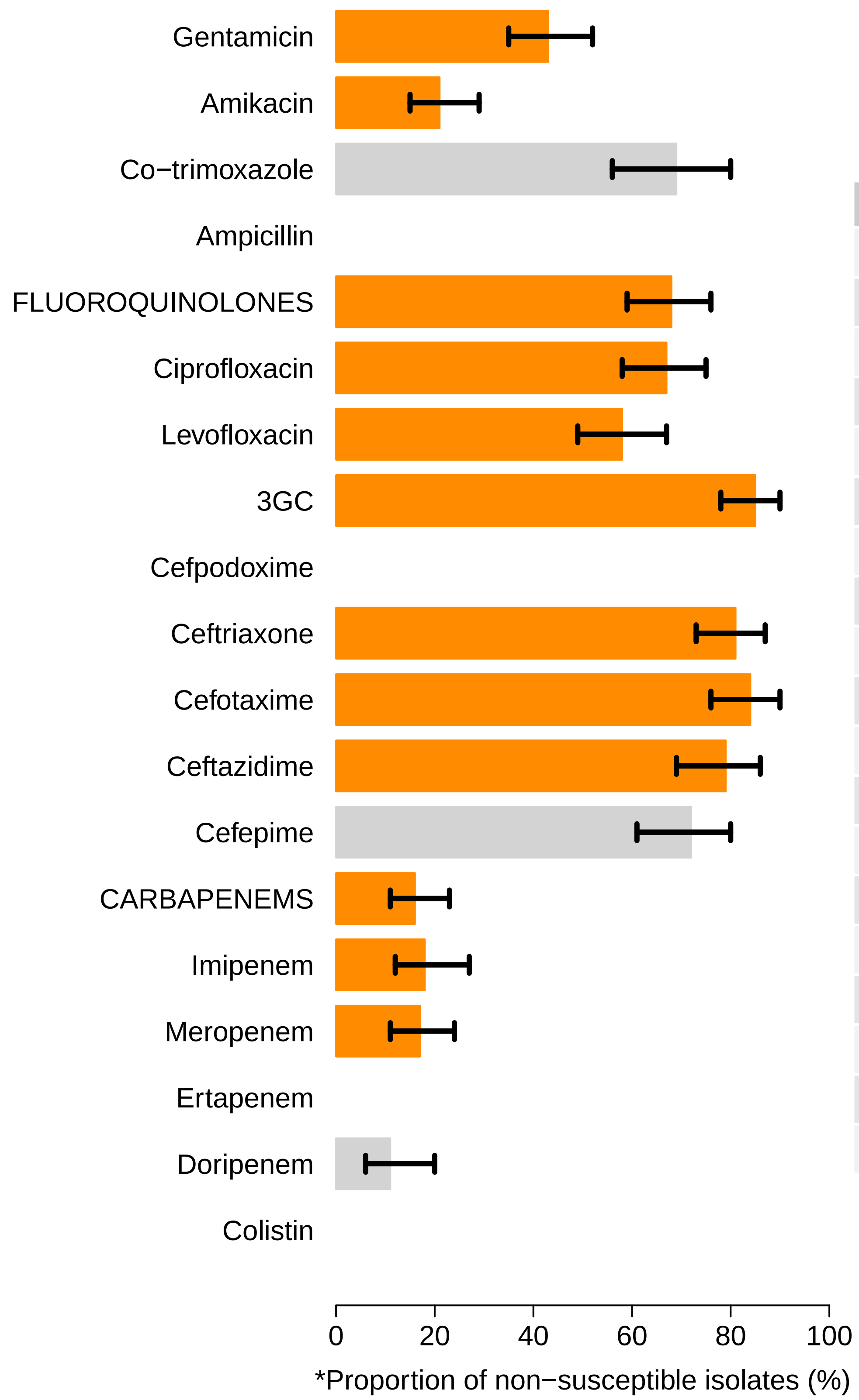

Antibiotic agent	% NS (n)	95% CI
Gentamicin	43% (52/120)	35%−52%
Amikacin	21% (26/125)	15%−29%
Co−trimoxazole	69% (38/55)	56%−80%
Ampicillin	NA	−
FLUOROQUINOLONES	68% (81/119)	59%−76%
Ciprofloxacin	67% (78/117)	58%−75%
Levofloxacin	58% (63/108)	49%−67%
3GC	85% (111/131)	78%−90%
Cefpodoxime	NA	−
Ceftriaxone	81% (91/112)	73%−87%
Cefotaxime	84% (89/106)	76%−90%
Ceftazidime	79% (77/98)	69%−86%
Cefepime	72% (61/85)	61%−80%
CARBAPENEMS	16% (21/132)	11%−23%
Imipenem	18% (19/103)	12%−27%
Meropenem	17% (19/115)	11%−24%
Ertapenem	NA	−
Doripenem	11% (9/82)	6%−20%
Colistin	NA	−

*Proportion of non−susceptible isolates (% NS) represents the number of patients with blood culture positive for non−susceptible isolates (numerator) over the total number of patients with blood culture positive for the organism and the organism was tested for susceptibility against the antibiotic (denominator). The AMASS application de−duplicated the data by including only the first isolate per patient per specimen type per evaluation period. Grey bars indicate that testing with the antibiotic occurred for less than 70% of the total number of blood culture positive for the organism.
CI = confidence interval; NA = Not available/reported/tested; 3GC = 3rd−generation cephalosporin; FLUOROQUINOLONES: ciprofloxacin or levofloxacin; CARBAPENEMS: imipenem, meropenem, ertapenem or doripenem

Section [3]: Isolate−based surveillance report with stratification by infection origin

Blood: ***Klebsiella pneumoniae*** **Community−origin** ***(No. of patients = 65)***

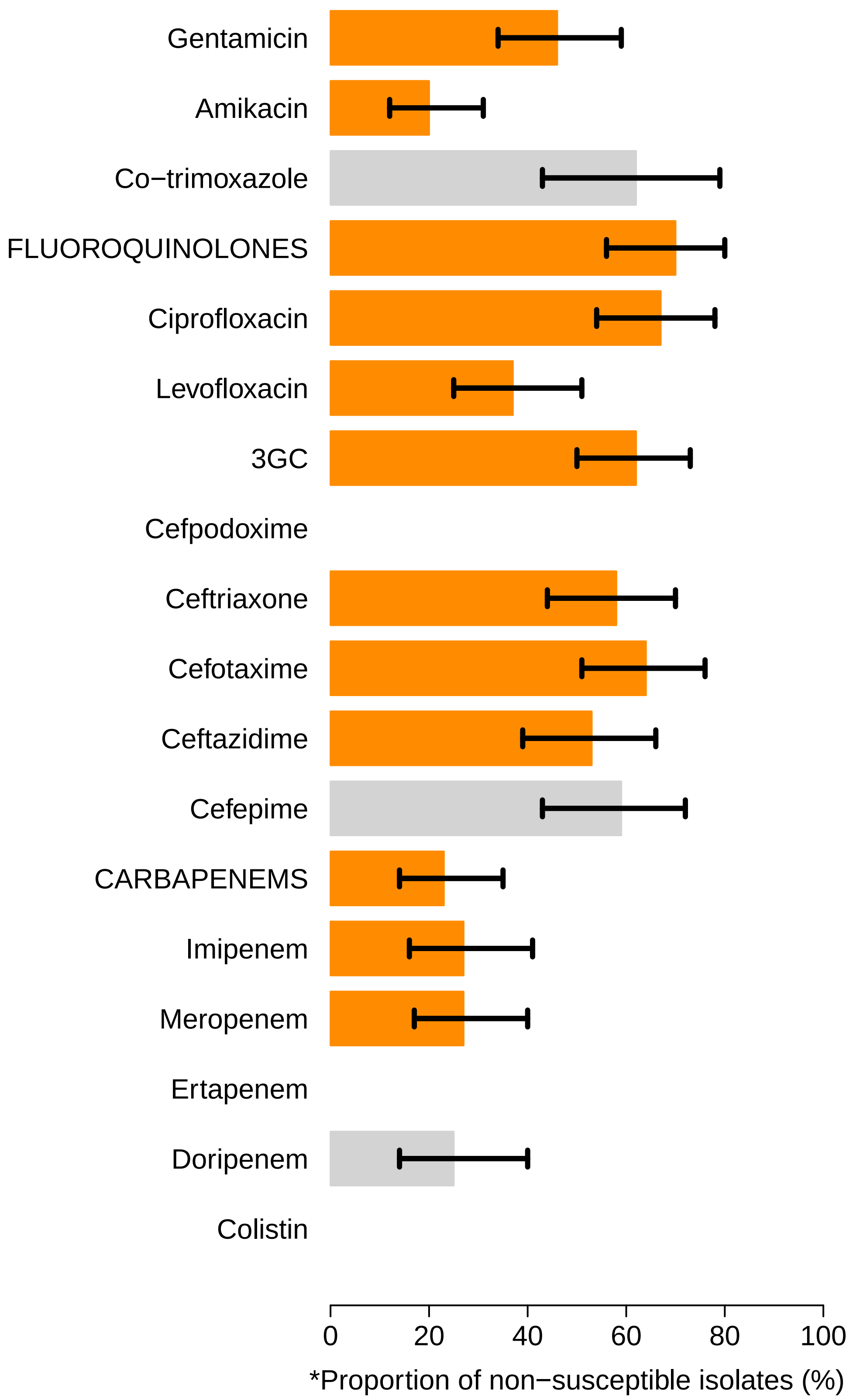

Antibiotic agent	% NS (n)	95% CI
Gentamicin	46% (26/56)	34%−59%
Amikacin	20% (12/61)	12%−31%
Co−trimoxazole	62% (15/24)	43%−79%
FLUOROQUINOLONES	70% (37/53)	56%−80%
Ciprofloxacin	67% (35/52)	54%−78%
Levofloxacin	37% (18/49)	25%−51%
3GC	62% (40/64)	50%−73%
Cefpodoxime	NA	−
Ceftriaxone	58% (30/52)	44%−70%
Cefotaxime	64% (34/53)	51%−76%
Ceftazidime	53% (26/49)	39%−66%
Cefepime	59% (24/41)	43%−72%
CARBAPENEMS	23% (14/61)	14%−35%
Imipenem	27% (12/45)	16%−41%
Meropenem	27% (14/52)	17%−40%
Ertapenem	NA	−
Doripenem	25% (10/40)	14%−40%
Colistin	NA	−

*Proportion of non−susceptible isolates (% NS) represents the number of patients with blood culture positive for non−susceptible isolates (numerator) over the total number of patients with blood culture positive for the organism and the organism was tested for susceptibility against the antibiotic (denominator). The AMASS application de−duplicated the data by including only the first isolate per patient per specimen type per evaluation period. Grey bars indicate that testing with the antibiotic occurred for less than 70% of the total number of blood culture positive for the organism.
CI = confidence interval; NA = Not available/reported/tested; 3GC = 3rd−generation cephalosporin; FLUOROQUINOLONES: ciprofloxacin or levofloxacin; CARBAPENEMS: imipenem, meropenem, ertapenem or doripenem

Section [3]: Isolate−based surveillance report with stratification by infection origin

Blood: ***Klebsiella pneumoniae*** **Hospital−origin** ***(No. of patients = 376)***

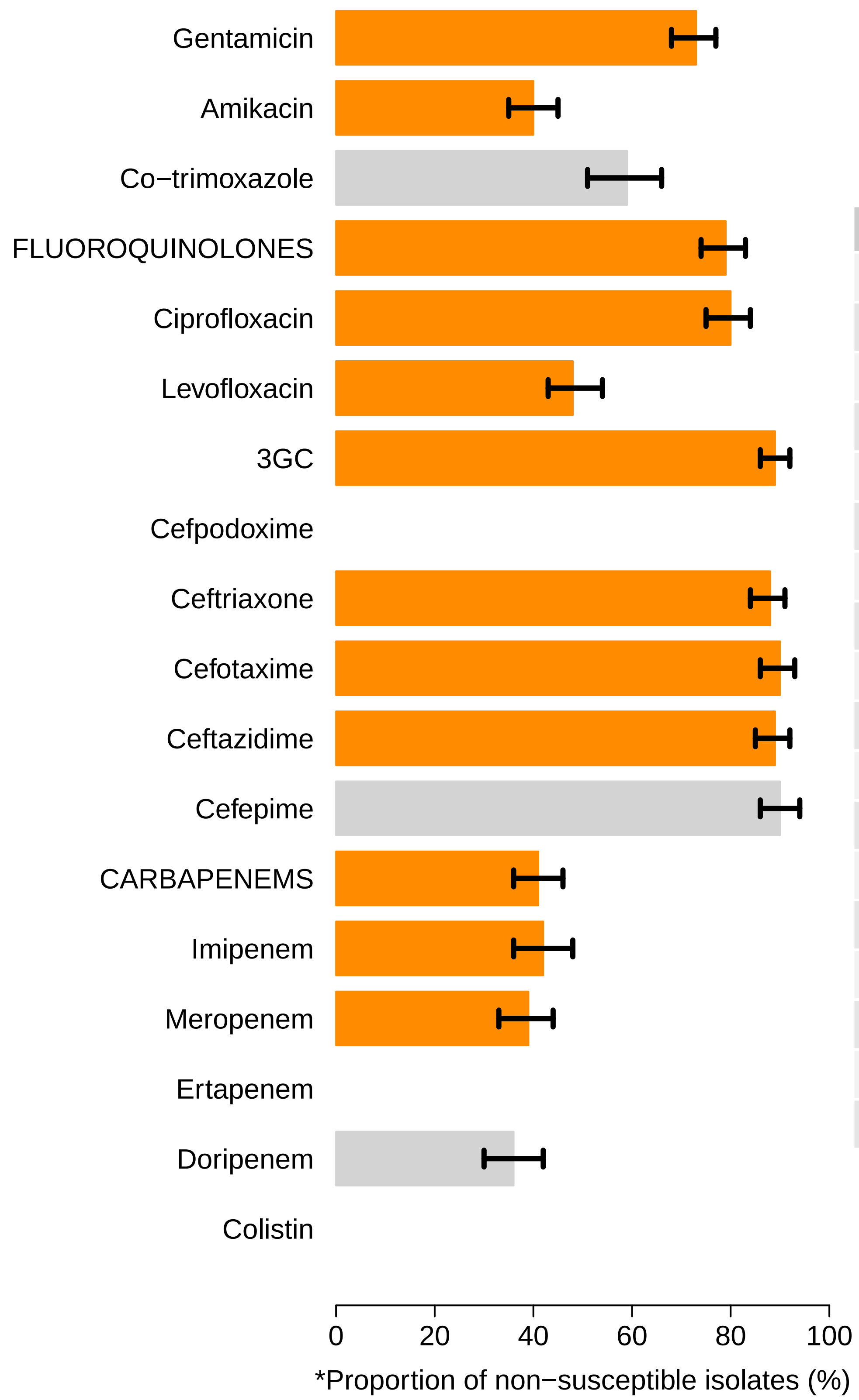

Antibiotic agent	% NS (n)	95% CI
Gentamicin	73% (242/332)	68%−77%
Amikacin	40% (136/341)	35%−45%
Co−trimoxazole	59% (100/170)	51%−66%
FLUOROQUINOLONES	79% (254/323)	74%−83%
Ciprofloxacin	80% (244/306)	75%−84%
Levofloxacin	48% (146/302)	43%−54%
3GC	89% (331/370)	86%−92%
Cefpodoxime	NA	−
Ceftriaxone	88% (280/317)	84%−91%
Cefotaxime	90% (252/280)	86%−93%
Ceftazidime	89% (251/281)	85%−92%
Cefepime	90% (216/239)	86%−94%
CARBAPENEMS	41% (145/358)	36%−46%
Imipenem	42% (113/271)	36%−48%
Meropenem	39% (119/307)	33%−44%
Ertapenem	NA	−
Doripenem	36% (87/243)	30%−42%
Colistin	NA	−

*Proportion of non−susceptible isolates (% NS) represents the number of patients with blood culture positive for non−susceptible isolates (numerator) over the total number of patients with blood culture positive for the organism and the organism was tested for susceptibility against the antibiotic (denominator). The AMASS application de−duplicated the data by including only the first isolate per patient per specimen type per evaluation period. Grey bars indicate that testing with the antibiotic occurred for less than 70% of the total number of blood culture positive for the organism.
CI = confidence interval; NA = Not available/reported/tested; 3GC = 3rd−generation cephalosporin; FLUOROQUINOLONES: ciprofloxacin or levofloxacin; CARBAPENEMS: imipenem, meropenem, ertapenem or doripenem

Section [3]: Isolate−based surveillance report with stratification by infection origin

Blood: *Pseudomonas aeruginosa* Community−origin ***(No. of patients = 60)***

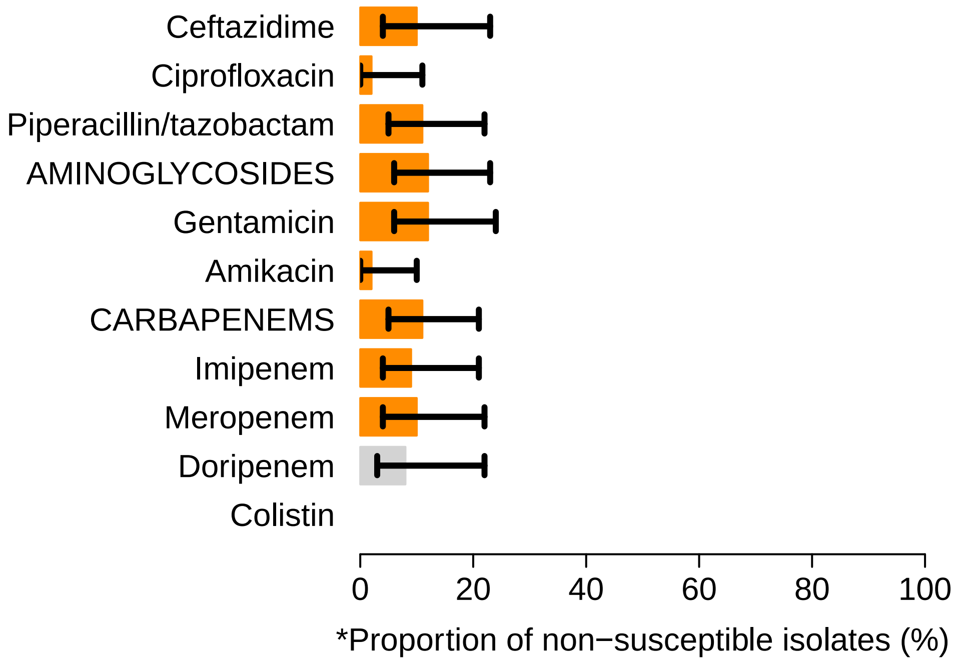

Antibiotic agent	% NS (n)	95% CI
Ceftazidime	10% (4/40)	4%−23%
Ciprofloxacin	2% (1/46)	0%−11%
Piperacillin/tazobactam	11% (6/55)	5%−22%
AMINOGLYCOSIDES	12% (7/57)	6%−23%
Gentamicin	12% (6/49)	6%−24%
Amikacin	2% (1/54)	0%−10%
CARBAPENEMS	11% (6/57)	5%−21%
Imipenem	9% (4/45)	4%−21%
Meropenem	10% (5/49)	4%−22%
Doripenem	8% (3/36)	3%−22%
Colistin	NA	−

Blood: *Pseudomonas aeruginosa* Hospital−origin ***(No. of patients = 100)***

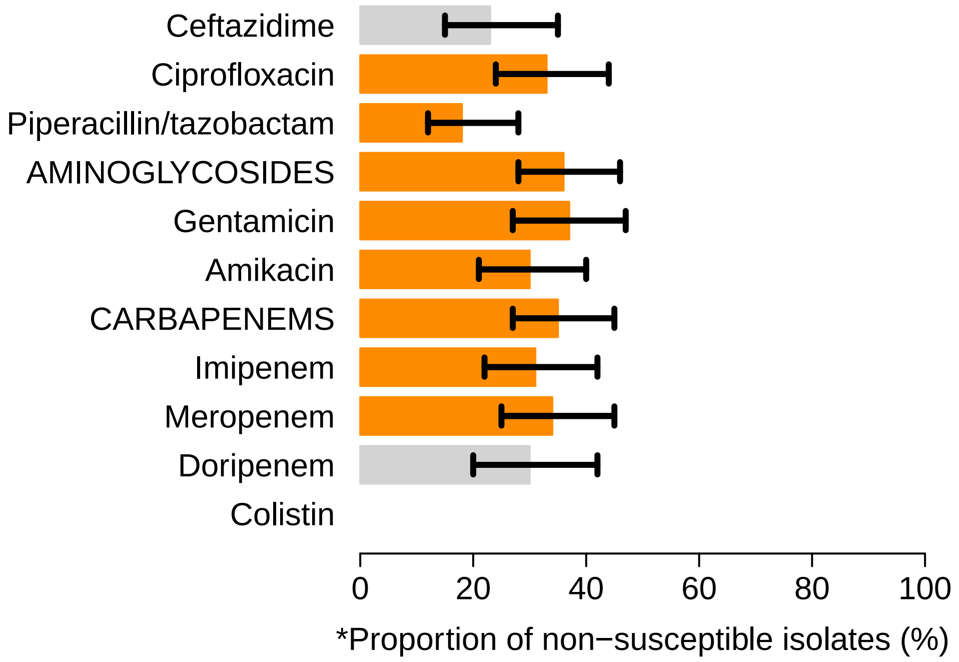

Antibiotic agent	% NS (n)	95% CI
Ceftazidime	23% (15/65)	15%−35%
Ciprofloxacin	33% (26/79)	24%−44%
Piperacillin/tazobactam	18% (17/92)	12%−28%
AMINOGLYCOSIDES	36% (35/96)	28%−46%
Gentamicin	37% (32/87)	27%−47%
Amikacin	30% (27/91)	21%−40%
CARBAPENEMS	35% (34/96)	27%−45%
Imipenem	31% (24/77)	22%−42%
Meropenem	34% (28/82)	25%−45%
Doripenem	30% (18/61)	20%−42%
Colistin	NA	−

*Proportion of non−susceptible isolates (% NS) represents the number of patients with blood culture positive for non−susceptible isolates (numerator) over the total number of patients with blood culture positive for the organism and the organism was tested for susceptibility against the antibiotic (denominator). The AMASS application de−duplicated the data by including only the first isolate per patient per specimen type per evaluation period. Grey bars indicate that testing with the antibiotic occurred for less than 70% of the total number of blood culture positive for the organism.
CI = confidence interval; NA = Not available/reported/tested; AMINOGLYCOSIDES: either gentamicin or amikacin; CARBAPENEMS: imipenem, meropenem, ertapenem or doripenem

Section [3]: Isolate−based surveillance report with stratification by infection origin

Blood: *Acinetobacter* spp. Community−origin (*No. of patients = 53*)

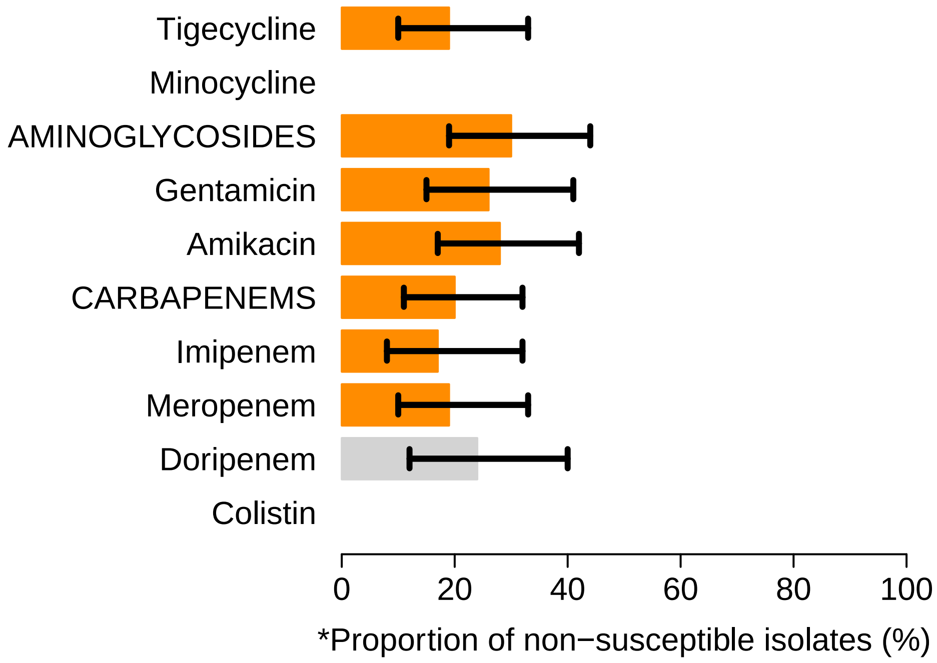

Antibiotic agent	% NS (n)	95% CI
Tigecycline	19% (9/47)	10%−33%
Minocycline	NA	−
AMINOGLYCOSIDES	30% (15/50)	19%−44%
Gentamicin	26% (11/42)	15%−41%
Amikacin	28% (13/47)	17%−42%
CARBAPENEMS	20% (10/51)	11%−32%
Imipenem	17% (6/36)	8%−32%
Meropenem	19% (9/47)	10%−33%
Doripenem	24% (8/34)	12%−40%
Colistin	NA	−

Blood: *Acinetobacter* spp. Hospital−origin (*No. of patients = 196*)

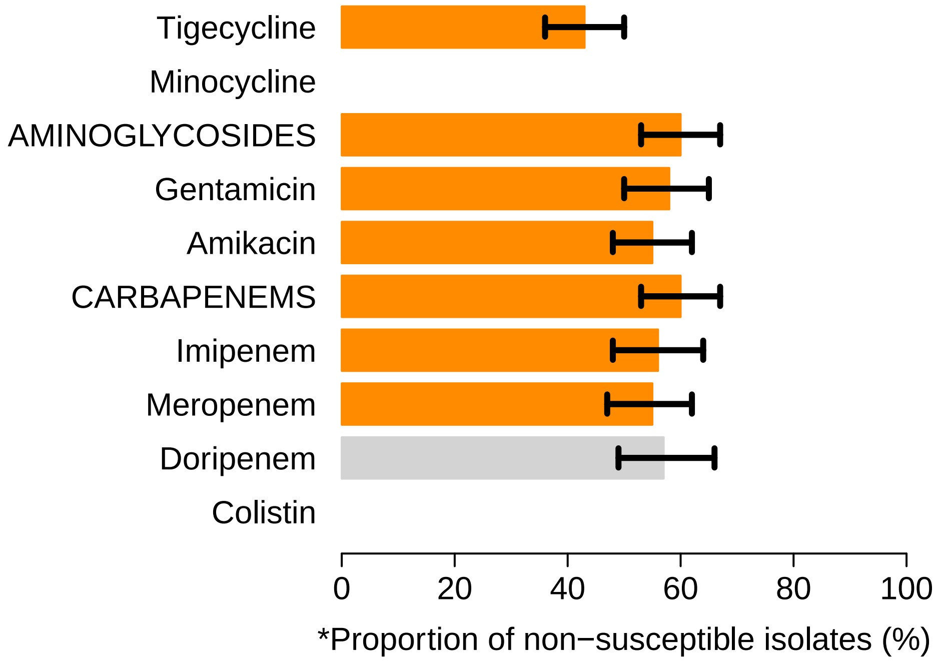

Antibiotic agent	% NS (n)	95% CI
Tigecycline	43% (74/173)	36%−50%
Minocycline	NA	−
AMINOGLYCOSIDES	60% (110/182)	53%−67%
Gentamicin	58% (96/166)	50%−65%
Amikacin	55% (95/172)	48%−62%
CARBAPENEMS	60% (110/182)	53%−67%
Imipenem	56% (79/140)	48%−64%
Meropenem	55% (84/154)	47%−62%
Doripenem	57% (69/120)	49%−66%
Colistin	NA	−

*Proportion of non−susceptible isolates (% NS) represents the number of patients with blood culture positive for non−susceptible isolates (numerator) over the total number of patients with blood culture positive for the organism and the organism was tested for susceptibility against the antibiotic (denominator). The AMASS application de−duplicated the data by including only the first isolate per patient per specimen type per evaluation period. Grey bars indicate that testing with the antibiotic occurred for less than 70% of the total number of blood culture positive for the organism.
CI = confidence interval; NA = Not available/reported/tested; AMINOGLYCOSIDES: either gentamicin or amikacin; CARBAPENEMS: imipenem, meropenem, ertapenem or doripenem

Section [4]: Sample−based surveillance report

Introduction

A sample−based surveillance report is generated if data of culture negative is available.

The sample−based approach involves the collection of data on all blood samples taken for microbiological testing and includes information on the number of positive blood samples for a specific specimen type (both pathogens under the survey and other bacteria) as well as number of negative (no microbial growth) samples. After removal of duplicate results and assuming that routine blood culture testing is applied systematically, we can use the number of tested patients as a proxy for a number of patients with new cases of bloodstream infection (BSI).

Results:

The microbiology_data file had:

Specimen collection dates ranged from ***01 Jan 2019 to 31 Dec 2020***

Number of records on blood specimen collected within the above date range:

17901 blood specimen records

**Number of patients sampled for blood culture within the above date range:*

8175 patients sampled for blood culture

Note:

*Number of patients sampled for blood culture is used as denominator to estimate the frequency of infections per 100,000 tested patients

The following figures show the frequncy of infections for patients with blood culture tested.

Section [4]: Sample−based surveillance report

Blood: ***Pathogens under this surveillance** ***(No. of patients = 8175)***

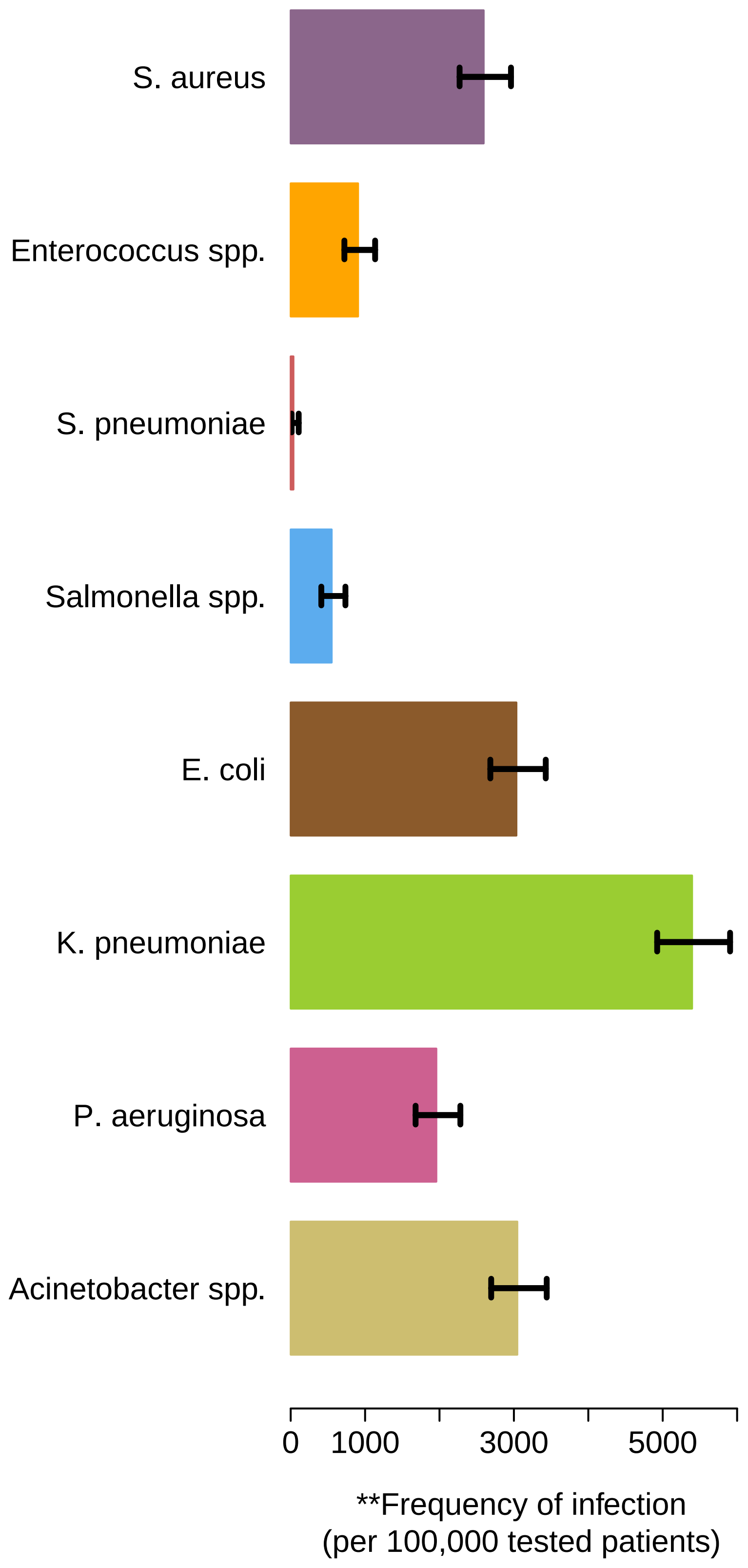

Organisms	**Frequency (95% CI)
S. aureus	2594 (2271−2961)
Enterococcus spp.	906 (722−1135)
S. pneumoniae	37 (13−108)
Salmonella spp.	551 (412−736)
E. coli	3034 (2684−3429)
K. pneumoniae	5395 (4926−5906)
P. aeruginosa	1958 (1679−2281)
Acinetobacter spp.	3046 (2695−3442)

*We apologise that the bacteria name in the table and in the figure are not written in italic. This is because of the R command we used. We will improve this in the next version.
**Frequency of infection per 100,000 tested patients represents the number of patients with blood culture positive for a pathogen (numerator) over the total number of tested patients (denominator). The AMASS application de−duplicates the data by included only the first isolate of each patient per specimen type per reporting period.
CI = confidence interval; NS = non−susceptible; NA = Not available/reported/tested; 3GC = 3rd−generation cephalosporin

Section [4]: Sample−based surveillance report

Blood: ***AMR pathogens under this surveillance** ***(No. of patients = 8175)***

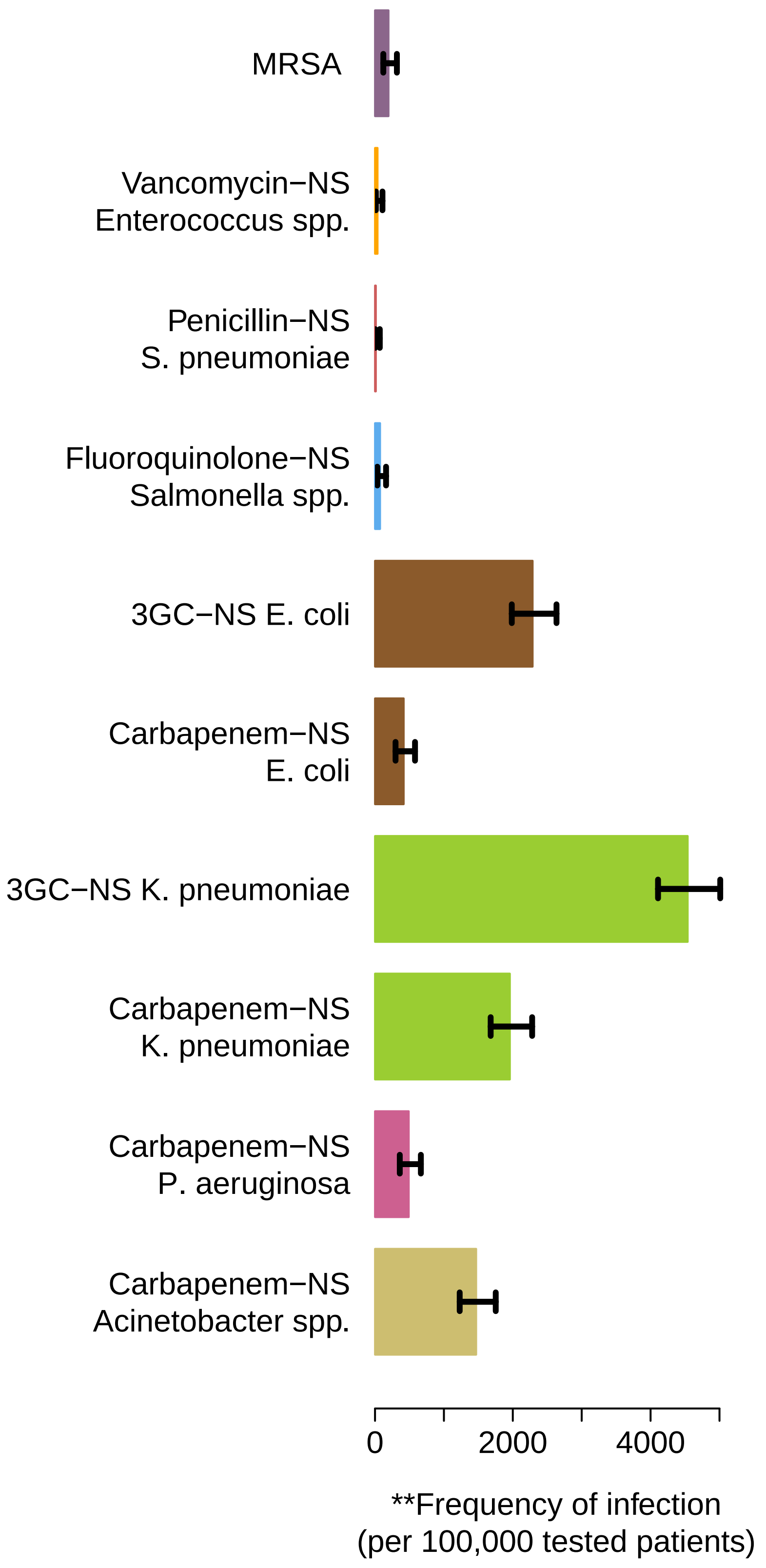

Organism	**Frequency (95% CI)
MRSA	196 (121−318)
Vancomycin−NS Enterococcus spp.	37 (13−108)
Penicillin−NS S. pneumoniae	13 (3−70)
Fluoroquinolone−NS Salmonella spp.	74 (34−161)
3GC−NS E. coli	2288 (1986−2635)
Carbapenem−NS E. coli	416 (298−581)
3GC−NS K. pneumoniae	4539 (4108−5012)
Carbapenem−NS K. pneumoniae	1958 (1679−2281)
Carbapenem−NS P. aeruginosa	490 (360−666)
Carbapenem−NS Acinetobacter spp.	1468 (1230−1753)

*We apologise that the bacteria name in the table and in the figure are not written in italic. This is because of the R command we used. We will improve this in the next version.
**Frequency of infection per 100,000 tested patients represents the number of patients with blood culture positive for a pathogen (numerator) over the total number of tested patients (denominator). The AMASS application de−duplicates the data by included only the first isolate of each patient per specimen type per reporting period.
CI = confidence interval; NS = non−susceptible; NA = Not available/reported/tested; 3GC = 3rd−generation cephalosporin

Section [5]: Sample−based surveillance report with stratification by infection origin

Introduction

A sample−based surveillance report with stratification by origin of infection is generated only if data of culture negative is available and admission date or a variable containing the classification is available in the raw data file with the appropriate specification in the data dictionaries.

Results:

The data included in the analysis had:

Specimen collection dates ranged from ***01 Jan 2019 to 31 Dec 2020***

Number of records on blood specimen collected within the above date range:

17901 blood specimen records

Number of patients sampled for blood culture within the above date range:

8175 patients sampled for blood culture

4128 *patients had at least one admission having the first blood culture drawn within first 2 calendar days of hospital admission.*

This parameter is used as a denominators for frequency of community−origin bacteraemia (per 100,000 patients tested for blood culture on admission).

4736 *patients had at least one admission having the first blood culture drawn after 2 calendar days of hospital admission.*

This parameter is used as a denominators for frequency of hospital−origin bacteraemia (per 100,000 patients tested for blood culture for HAI).

0 *patients had a blood drawn for culture and with unknown origin of infection.*

Validation of this statistics is highly recommended.

Note:

689 *patients had more than one admissions, of which at least one admission had the first blood culture drawn within the first 2 calendar days of hospital admission AND at least one admission had the first blood culture drawn after 2 calendar days of hospital admission.*

The following figures show the frequency of infections for patients with blood culture tested and stratified by infection origin, under this surveillance.

Section [5]: Sample−based surveillance report with stratification by infection origin

Blood: ***Pathogens** **Community−origin** ***(No. of patients = 4128)***

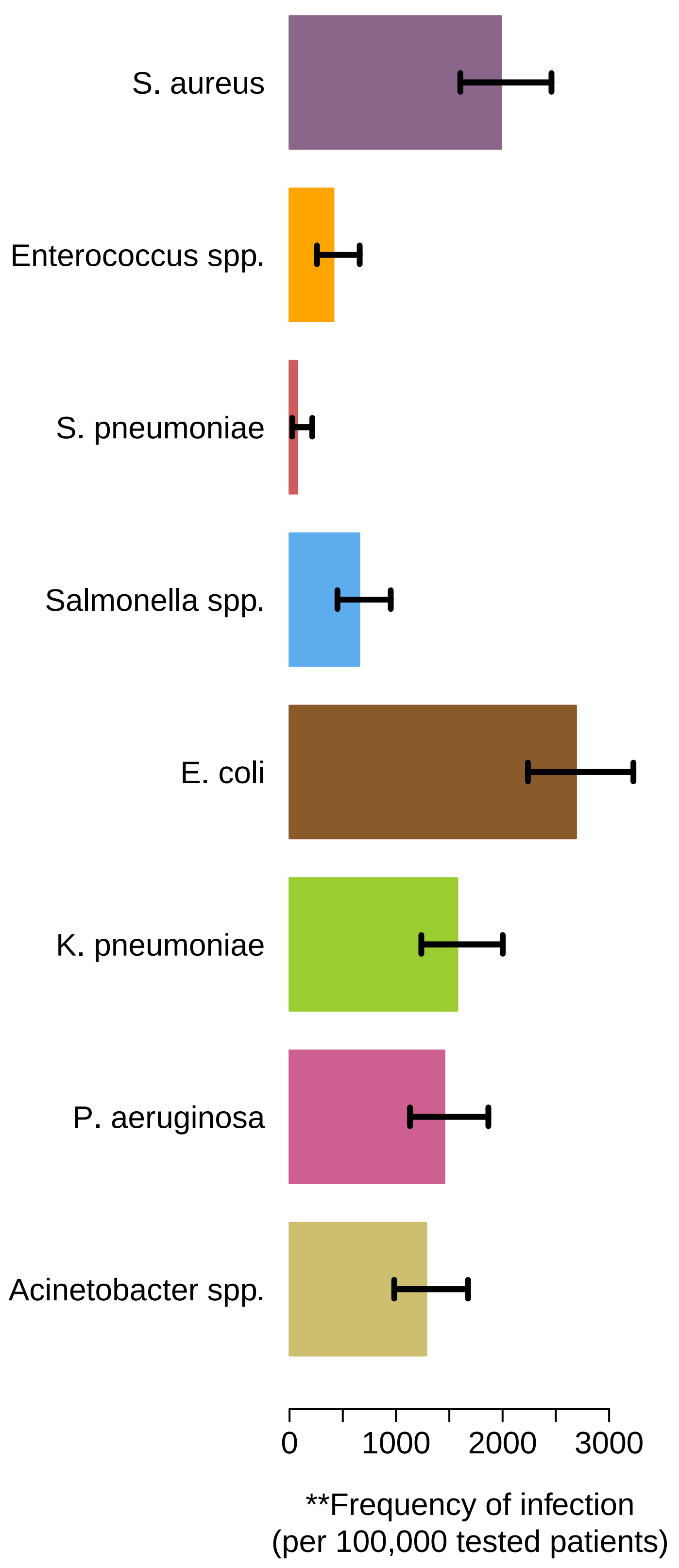

Organism	**Frequency (95% CI)
S. aureus	1987 (1604−2459)
Enterococcus spp.	412 (258−659)
S. pneumoniae	73 (25−214)
Salmonella spp.	655 (450−950)
E. coli	2689 (2238−3229)
K. pneumoniae	1575 (1238−2002)
P. aeruginosa	1454 (1131−1867)
Acinetobacter spp.	1284 (983−1676)

*We apologise that the bacteria name in the table and in the figure are not written in italic. This is because of the R command we used. We will improve this in the next version.

**Frequency of infection per 100,000 tested patients on admission represents the number of patients with blood culture positive for a pathogen (numerator) over the total number of tested population on admission (denominator). The AMASS application de−duplicates the data by included only the first isolate of each patient per specimen type per reporting period.

CI = confidence interval; NS = non−susceptible; NA = Not available/reported/tested; 3GC = 3rd−generation cephalosporin

Section [5]: Sample−based surveillance report with stratification by infection origin

Blood: ***Pathogens** **Hospital−origin** ***(No. of patients = 4736)***

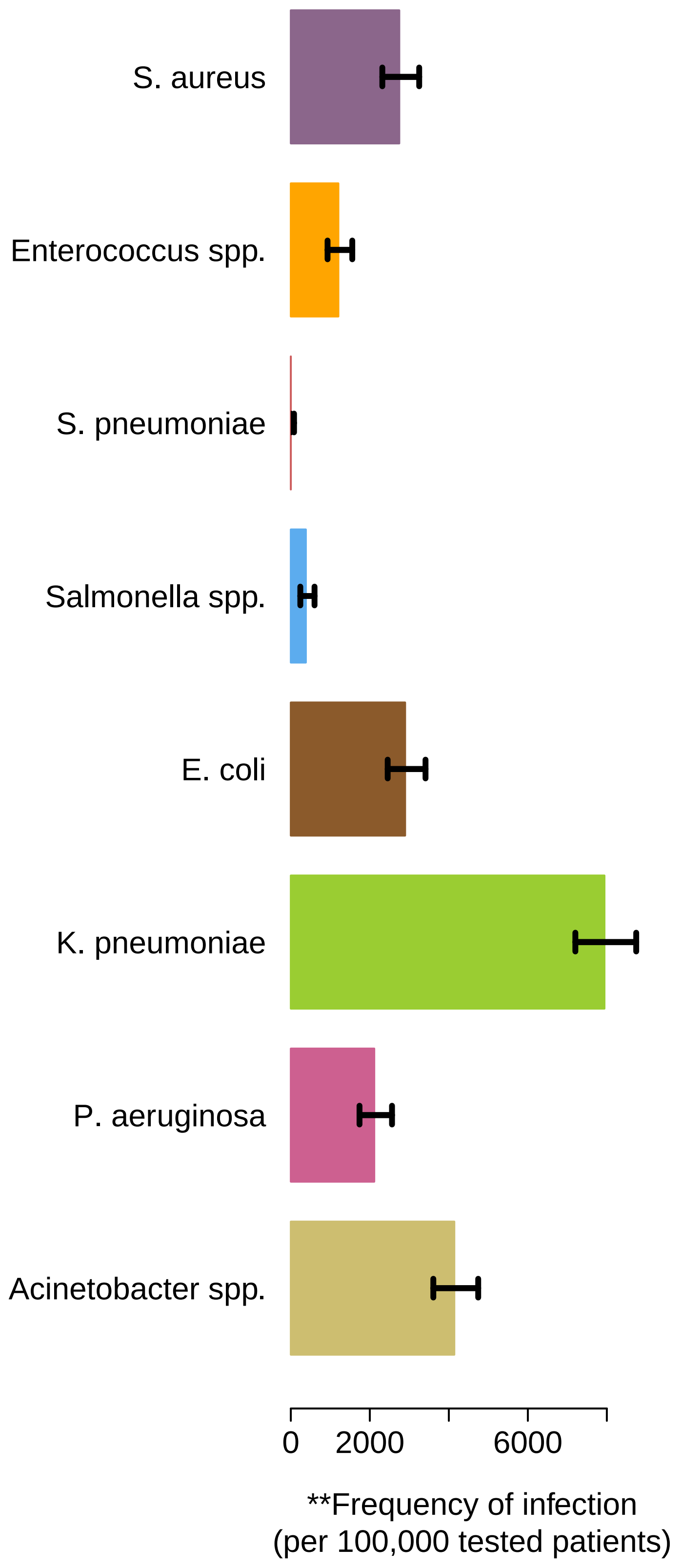

Organism	**Frequency (95% CI)
S. aureus	2745 (2317−3250)
Enterococcus spp.	1204 (931−1557)
S. pneumoniae	0 (0−82)
Salmonella spp.	381 (241−601)
E. coli	2893 (2453−3410)
K. pneumoniae	7940 (7203−8744)
P. aeruginosa	2112 (1740−2562)
Acinetobacter spp.	4139 (3608−4744)

*We apologise that the bacteria name in the table and in the figure are not written in italic. This is because of the R command we used. We will improve this in the next version.
**Frequency of infection per 100,000 tested population at risk of HAI represents the number of patients with blood culture positive for a pathogen (numerator) over the total number of tested population at risk of HAI (denominator). The AMASS application de−duplicates the data by included only the first isolate of each patient per specimen type per reporting period.
CI = confidence interval; NS = non−susceptible; NA = Not available/reported/tested; 3GC = 3rd−generation cephalosporin

Section [5]: Sample−based surveillance report with stratification by infection origin

Blood: ***AMR pathogens** **Community−origin** ***(No. of patients = 4128)***

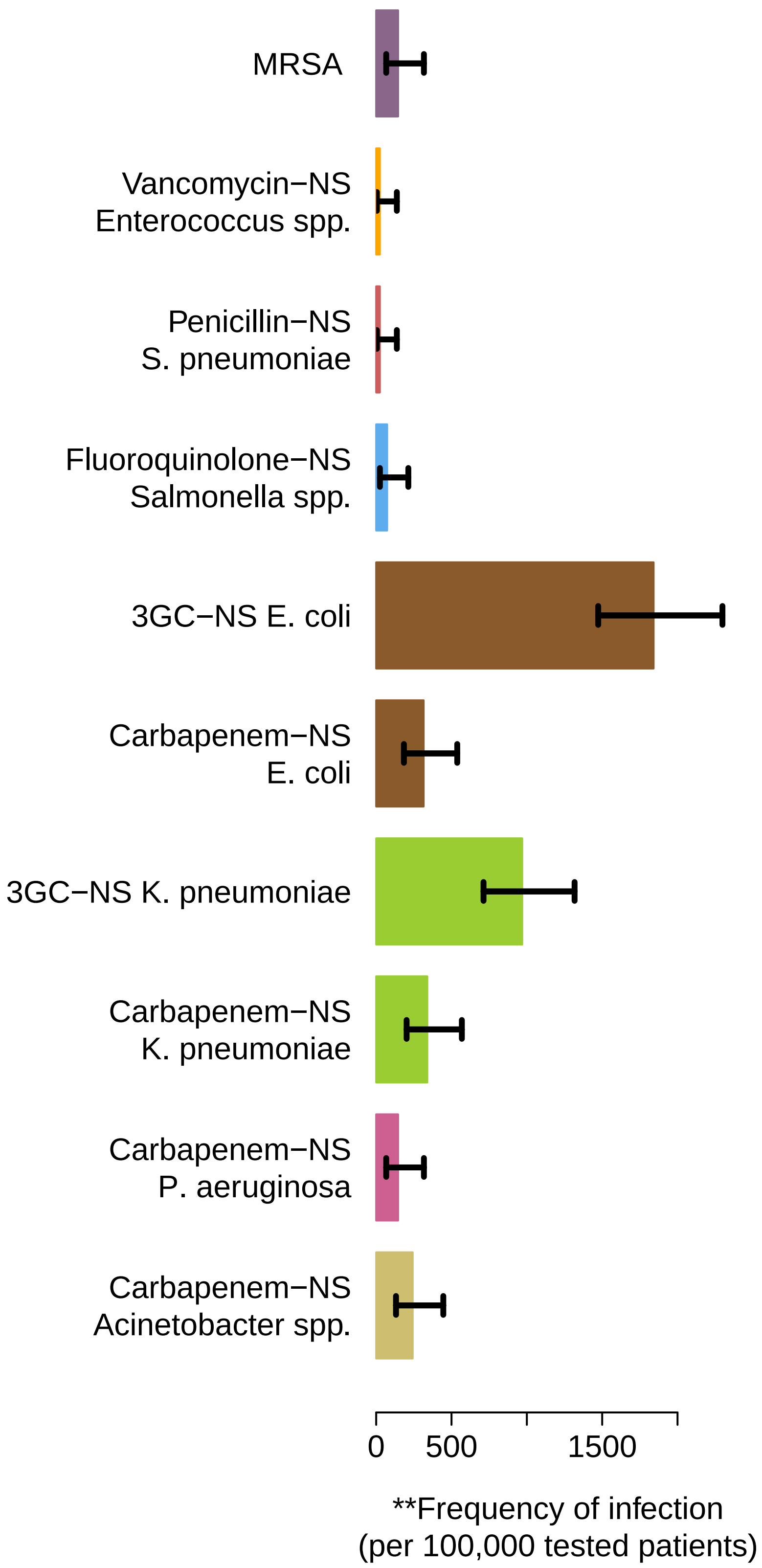

Organism	**Frequency (95% CI)
MRSA	146 (67−317)
Vancomycin−NS Enterococcus spp.	25 (5−138)
Penicillin−NS S. pneumoniae	25 (5−138)
Fluoroquinolone−NS Salmonella spp.	73 (25−214)
3GC−NS E. coli	1842 (1474−2299)
Carbapenem−NS E. coli	315 (185−539)
3GC−NS K. pneumoniae	969 (713−1317)
Carbapenem−NS K. pneumoniae	340 (203−569)
Carbapenem−NS P. aeruginosa	146 (67−317)
Carbapenem−NS Acinetobacter spp.	243 (132−446)

*We apologise that the bacteria name in the table and in the figure are not written in italic. This is because of the R command we used. We will improve this in the next version.
**Frequency of infection per 100,000 tested patients represents the number of patients with blood culture positive for a pathogen (numerator) over the total number of tested patients (denominator). The AMASS application de−duplicates the data by included only the first isolate of each patient per specimen type per reporting period.
CI = confidence interval; NS = non−susceptible; NA = Not available/reported/tested; 3GC = 3rd−generation cephalosporin

Section [5]: Sample−based surveillance report with stratification by infection origin

Blood: ***AMR pathogens** **Hospital−origin** ***(No. of patients = 4736)***

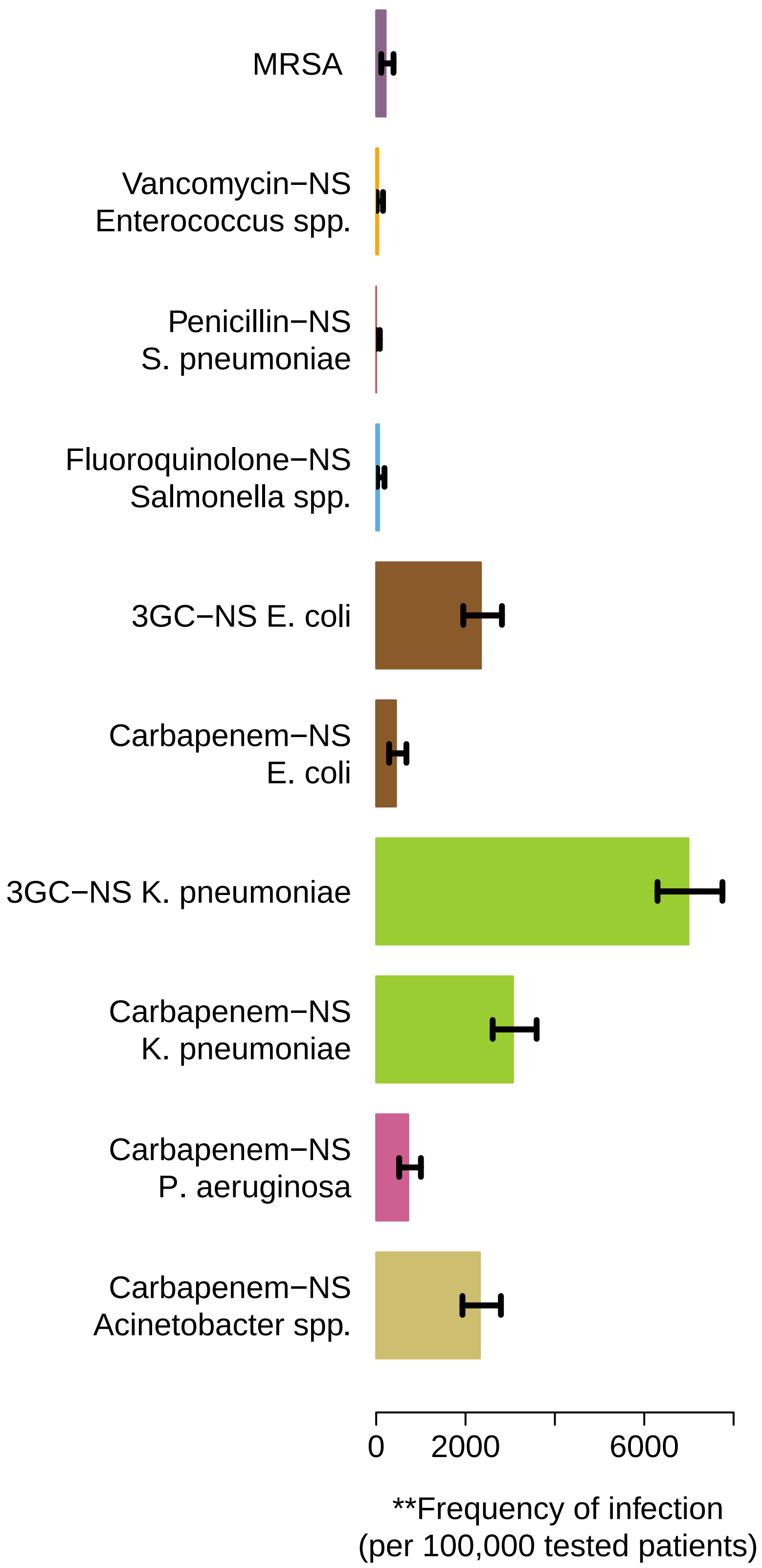

Organism	**Frequency (95% CI)
MRSA	212 (115−389)
Vancomycin−NS Enterococcus spp.	43 (12−154)
Penicillin−NS S. pneumoniae	0 (0−82)
Fluoroquinolone−NS Salmonella spp.	64 (22−187)
3GC−NS E. coli	2344 (1950−2815)
Carbapenem−NS E. coli	444 (291−677)
3GC−NS K. pneumoniae	6990 (6298−7751)
Carbapenem−NS K. pneumoniae	3062 (2608−3592)
Carbapenem−NS P. aeruginosa	718 (515−1002)
Carbapenem−NS Acinetobacter spp.	2323 (1931−2792)

*We apologise that the bacteria name in the table and in the figure are not written in italic. This is because of the R command we used. We will improve this in the next version.
**Frequency of infection per 100,000 tested patients represents the number of patients with blood culture positive for a pathogen (numerator) over the total number of tested patients (denominator). The AMASS application de−duplicates the data by included only the first isolate of each patient per specimen type per reporting period.
CI = confidence interval; NS = non−susceptible; NA = Not available/reported/tested; 3GC = 3rd−generation cephalosporin

Report [6] Mortality in AMR antimicrobial−susceptible infections

Not applicable because hospital_admission_data.csv file is not available,
or in−hospital outcome (in hospital_admission_data.csv file) is not available.

Methods used by the AMASS application

Data source:
For each run (double−click on AMASS.bat file), the AMASS application used the microbiology data file (microbiology_data) and the hospital admission data file (hospital_admission_data) that were stored in the same folder as the application file. Hence, if the user would like to update, correct, revise or change the data, the data files in the folder should be updated before the AMASS.bat file is double−clicked again. A new report based on the updated data would then be generated.

Requirements:
− Computer with Microsoft Windows 7 or 10
AMASS may work in other versions of Microsoft Windows and other operating systems. However, thorough testing and adjustment have not been performed.
− AMASS.zip package file
The AMASS application is to be downloaded from http://www.amass.website, and unzipped to generate an AMASS folder that could be stored under any folder in the computer. The AMASS folder contains 4 files (AMASS.bat, z_Rcode.R, dictionary_for_microbiology_data.xlsx, and dictionary_for_hospital_admission_data.xlsx), and 5 folders (Variables, Rprogram, Example_Dataset_1_WHONET, Example_Dataset_2, and ResultData).
− Microbiology data file (microbiology_data in .csv or .xlsx file format)
The user needs to obtain microbiology data, and then copy & paste this data file into the same folder as the AMASS.bat file.
− [Optional] Hospital admission data file (hospital_admission_data)
If available, the user could obtain hospital admission data, and then copy & paste this data file into the same folder as the AMASS.bat file.

Not required:
− Internet to run AMASS application
The AMASS application will run offline. No data are transferred while the application is running and reports are being generated; the reports are in PDF format (do not contain any patient identifier) and can be shared under the user's jurisdiction.
− R
The download package (AMASS.zip) included R portable and R libraries that the AMASS application requires. The user does not need to install any programme before using the AMASS. The user also does not have to uninstall R prgramme if the computer already has the R prgramme installed. The user does not need to know how to use R prgramme.

Note:
[1] Please ensure that the file names of microbiology data file (microbiology_data) and the hospital admission data file (hospital_admission_data) are identical to what is written here. Please make sure that all are lower−cases with an underscore '_' at each space.
[2] Please ensure that both microbiology and hospital admission data files have no empty rows before the row of the variable names (i.e. the variable names are the first row in both files).
[3] For the first run, an user may need to fill the data dictionary files to make sure that the AMASS application understands your variable names and values.

AMASS uses a tier−based approach. In cases when only the microbiology data file with the results of culture positive samples is available, only section one and two would be generated for users. Section three would be generated only when data on admission date are available. This is because these data are required for the stratification by origin of infection. Section four would be generated only when data of specimens with culture negative (no microbial growth) are available in the microbiology data. This is because these are required for the sample−based approach. Section five would be generated only when both data of specimens with culture negative and admission date are available. Section six would be generated only when mortality data are available.

Mortality was calculated from the number of in−hospital deaths (numerator) over the total number of patients with blood culture positive for the organism (denominator). Please note that this is the all−cause mortality calculated using the outcome data in the data file, and may not necessarily represent the mortality directly due to the infections.

How to use data dictionary files
In cases when variable names in the microbiology and hospital admission data files were not the same as the one that AMASS used, the data dictionary files could be edited.
The raw microbiology and hospital admission data files were to be left unchanged.
The data dictionary files provided could be edited and re−used automatically when the microbiology and hospital admission data files were updated and the AMASS.bat were to be double−clicked again (i.e. the data dictionary files would allow the user to re−analyze data files without the need to adjust variable names and data value again every time).

For example:
If variable name for 'hospital number' is written as 'hn' in the raw data file, the user would need to add 'hn' in the cell next to 'hospital_number'. If data value for blood specimens is defined by 'Blood−Hemoculture' in the raw data file, then the user would need to add 'Blood−Hemoculture' in the cell next to 'blood_specimen'.

Dictionary file (dictionary_for_microbiology_data.xlsx) may show up as in the table below:

**Variable names used in AMASS**	**Variable names used in your microbiology data file**	**Requirements**
Don't change values in this column, but you can add rows with similar values if you need	Change values in this column to represent how variable names are written in your raw microbiology data file	
hospital_number		Required
**Values described in AMASS**	**Values used in your microbiology data file**	**Requirements**
blood_specimen		Required

Please fill in your variable names as follows:

**Variable names used in AMASS**	**Variable names used in your microbiology data file**	**Requirements**
Don't change values in this column, but you can add rows with similar values if you need	Change values in this column to represent how variable names are written in your raw microbiology data file	
hospital_number	hn	Required
**Values described in AMASS**	**Values used in your microbiology data file**	**Requirements**
blood_specimen	Blood−Hemoculture	Required

Then, save the file. For every time the user double−clicked AMASS.bat, the application would know that the variable named 'hn' is similar to 'hospital_number' and represents the patient identifier in the analysis.

Organisms included in this report:

- − *Staphylococcus aureus*
- − *Enterococcus* spp.
- − *Streptococcus pneumoniae*
- − *Salmonella* spp.
- − *Escherichia coli*
- − *Klebsiella pneumoniae*
- − *Pseudomonas aeruginosa*
- − *Acinetobacter* spp.

The eight organisms and antibiotics included in the report were selected based on the global priority list of antibiotic resistant bacteria and Global Antimicrobial Resistance Surveillance System (GLASS) of WHO [1,2].

Definitions:

The definitions of infection origin proposed by the WHO GLASS was used [1]. In brief, community−origin bloodstream infection (BSI) was defined for patients in the hospital within the first two calendar days of admission when the first blood culture positive specimens were taken. Hospital−origin BSI was defined for patients in the hospital longer than the first calendar days of admission when the first blood culture positive specimens were taken. In cases when the user had additional data on infection origin defined by infection control team or based on referral data, the user could edit the data dictionary file (variable name 'infection_origin') and the AMASS application would use the data of that variable to stratify the data by origin of infection instead of the above definition. However, in cases when data on infection origin were not available (as in many hospitals in LMICs), the above definition would be calculated based on admission date and specimen collection date (with cutoff of 2 calendar days) and used to classify infections as community−origin or hospital−origin.

De−duplication:

When more than one blood culture was collected during patient management, duplicated findings of the same patient were excluded (de−duplicated). Only one result was reported for each patient per sample type (blood) and surveyed organisms (listed above). For example, if two blood cultures from the same patient had *E. coli*, only the first would be included in the report. If there was growth of *E. coli* in one blood culture and of *K. pneumoniae* in the other blood culture, then both results would be reported. One would be for the report on *E. coli* and the other one would be for the report on *K. pneumoniae.*

References:

[1] World Health Organization (2018) Global Antimicrobial Resistance Surveillance System (GLASS) Report. Early implantation 2016−2017. http://apps.who.int/iris/bitstream/handle/10665/259744/9789241513449−eng.pdf. (accessed on 3 Dec 2018)

[2] World Health Organization (2017) Global priority list of antibiotic−resistant bacteria to guide research, discovery, and development of new antibiotics. https://www.who.int/medicines/publications/WHO−PPL−Short_Summary_25Feb−ET_NM_WHO.pdf. (accessed on 3 Dec 2018)

Investigator team

The AMASS application is being developed by Cherry Lim, Clare Ling, Elizabeth Ashley, Paul Turner, Rahul Batra, Rogier van Doorn, Soawapak Hinjoy, Sopon Iamsirithaworn, Susanna Dunachie, Tri Wangrangsimakul, Viriya Hantrakun, William Schilling, John Stelling, Jonathan Edgeworth, Guy Thwaites, Nicholas PJ Day, Ben Cooper and Direk Limmathurotskul.

The AMASS application was funded by the Wellcome Trust (grant no. 206736 and 101103). C.L. is funded by a Training Research Fellowship (grant no. 206736) and D.L. is funded by an Intermediate Training Fellowship (grant no. 101103) from the Wellcome Trust.
